# Correction: Developing an assistive technology usability questionnaire for people with neurological diseases

**DOI:** 10.1371/journal.pone.0308511

**Published:** 2024-08-02

**Authors:** Maria Masbernat-Almenara, Francesc Rubi-Carnacea, Eloy Opisso, Esther Duarte-Oller, Josep Medina-Casanovas, Fran Valenzuela-Pascual

There are errors in the Supporting Information Files. S2 File should appear as S3 File and the S2 File should have been the NATU questionnaire in Spanish version. Please see the correct S2 and S3 Files below.

## Supporting information

S2 File (PDF)

S3 File (XLSX)
