# Supplementary material for: Developing an assistive technology usability questionnaire for people with neurological diseases
Source: PLoS One. 2023 Jan 31;18(1):e0281197. doi: 10.1371/journal.pone.0281197 (PMC9888697; doi:10.1371/journal.pone.0281197)
Supplement: S1 File — (PDF) [file pone.0281197.s001.pdf]

S1 File. NATU Questionnaire, scoring and interpretation.

NATU Quest - English version

Product that I will assess:

………………………………………… (Please add the name of the product)

Please answer the questions from 0 to 5:

- 0 is Strongly Disagree
- 5 is Strongly Agree

1. I believe that …….. can help me improving my functional independence.

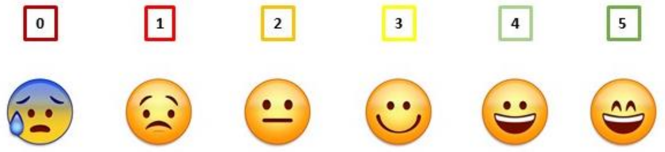

Comments:

2. I feel comfortable wearing/using …………………………..

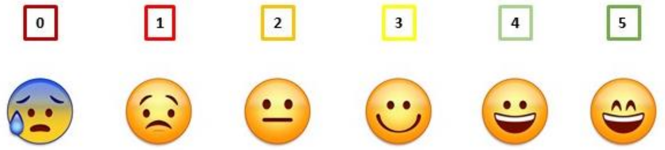

Comments:

3. ……………………..adapts to my characteristics and needs.

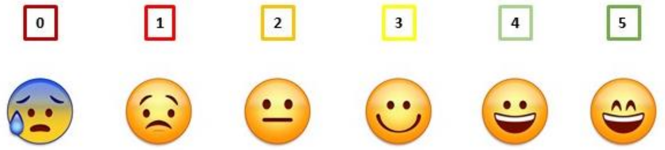

Comments:

4. Donning/Doffing …………….. is quick and easy for me.

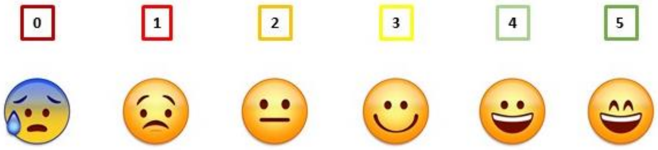

Comments:

5. I feel safe using/wearing ……………..// ………………… is safe in its use.

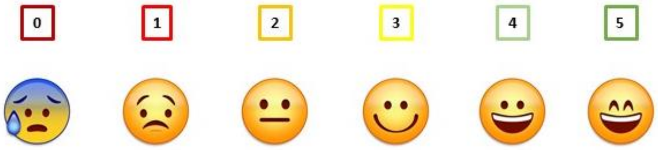

Comments:

6. ……………………….. allows me to achieve my goal / allows me to perform a movement/action that I could not do before

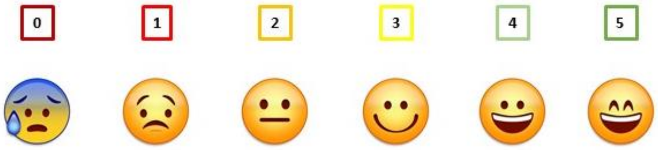

Comments:

7. ……………………………. adapts to me on my daily life needs

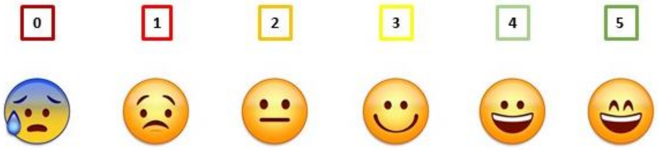

Comments:

8. In general, ………………………………. is easy to use.

0 1 2 3 4 5

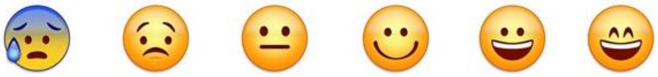

Comments:

9. Information and instructions of use …………………………. are easy to understand and easy to remember

0 1 2 3 4 5

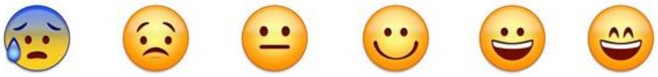

Comments:

10. Overall, I am satisfied with …………………

0 1 2 3 4 5

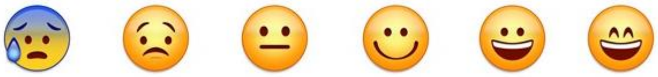

Comments:

Results:

☐ TOTAL

☐ Usable
☐ Slighlty Usable
☐ No usable

☐ Efficacy
☐ Comfortable
☐ Adaptability
☐ Easy to put on / off
☐ Safe
☐ Functionality
☐ Ergonomics
☐ Easy to use
☐ Easy to remember how to use it
☐ Satisfaction

Questionnaire score and interpretation

The questionnaire's total score was obtained by adding the scores of valid answers from 1 to 10 and dividing the sum by the number of valid items. This value varies from 0 to 5.

Result interpretation: Since the questionnaire is a 6 Likert scale the values from 0 to 2 are negative results and from 3 to 5 are positive results, following this premises:

- *Usable*. If the total score was 4–5 indicates that the user is satisfied with the product and is likely to use it. Also, if the product was a prototype or in development, the result indicated that the device meets the minimum requirements to be usable and does not need revision or needs very little revision of some items.
- *Slightly usable*. If the result was ≥ 3, it means that the user is not completely satisfied and may stop using it. Also, if the product was in development, it means that the device meets the minimum requirements to be usable but requires revision of some items.
- *No usable*. If the result was < 3, it means that the user is not completely satisfied, and inevitability will stop using it. Also, if the product is in development, it indicates that the device does not meet the minimum requirements to be usable and requires revision of several items or needs to be reconsidered.

It is possible to obtain a score for each item in order to provide a more precise indication of which items need an improvement in case that a new assistive technology is in developing. To calculated it, sum all the valid scores of each item of all the answered questionnaires and dividing the sum by the number of valid answers of all the questionnaires, hence an average score for each item was obtained.

- If the score obtained was 5, indicates that the aspect of the product addressed no require any revision.
- If the score obtained was < 5 and ≥ 3, indicates that this aspect of the product requires revision.
- If the score was < 3, this aspect of the product requires comprehensive revision.
