# Supplementary material for: Developing an assistive technology usability questionnaire for people with neurological diseases
Source: PLoS One. 2023 Jan 31;18(1):e0281197. doi: 10.1371/journal.pone.0281197 (PMC9888697; doi:10.1371/journal.pone.0281197)
Supplement: Attachment — Submitted filename: PONE-D-22-13676 with reviewers comments.pdf [file pone.0281197.s004.pdf]

PLOS ONE

<p class="Articletitle">Developing An Assistive Technology Usability Questionnaire for People with Neurological Diseases <o:p></o:p></p>

--Manuscript Draft--

**Manuscript Number:**	PONE-D-22-13676
**Article Type:**	Research Article
**Full Title:**	<p class="Articletitle">Developing An Assistive Technology Usability Questionnaire for People with Neurological Diseases <o:p></o:p></p>
**Short Title:**	Assistive Technology Usability Questionnaire for people with Neurological Diseases
**Corresponding Author:**	Francesc Rubí-Carnacea<br>University of Lleida: Universitat de Lleida<br>Lleida, Lleida SPAIN
**Keywords:**	Nervous System Diseases, Neurological Rehabilitation, Questionnaire Design, Self-Help Devices, User-centered Design
**Abstract:**	Purpose. This study describes the development of a questionnaire for assessing the usability of assistive technologies accessible to people with neurological diseases. Methods. A Delphi study was conducted to identify relevant items for the questionnaire. After that, the content validity was addressed to identify the essential items. Once the questionnaire was designed following the results of Delphi study and content validity, the reliability and validity, and the rasch model of the questionnaire were examined. Results . Two rounds of the Delphi study were carried out. A total 73 participants (42 experts and 31 users) participated in round 1 and 59 people (27 experts and 32 users) in round 2. A total of 53 and 29 items were identified in rounds 1 and 2, respectively. In the content validity we found nine items above the threshold of 0.58. Finally, 10 items were included in the questionnaire. 51 participants participate in the reliability and validity of the questionnaire. Internal consistency reliability of the questionnaire analyzed by Cronbach's Alpha was α=0,895. There was moderate to considerable concordance among our questionnaire items test-retest in the Kappa coefficient and a strong association between test-retest in the Spearman's coefficient ρ=0.818 (p<0,001). The intraclass correlation coefficient was 0,869 with 95% of confidence interval (0,781;0,923). There was a strong correlation of the total scores between the new questionnaire and other validate questionnaire analyzed with Spearman's coefficient ρ=0.756 (p<0,001). The 10 items demonstrated a satisfactory fit to the Rasch model. Conclusions . The present study suggested that the new questionnaire is a reliable 10-item usability questionnaire that allows subjective and quick assessment of the usability of assistive technologies by people with neurological diseases.
**Order of Authors:**	Maria Masbernat-Almenara
	Francesc Rubí-Carnacea
	Eloy Opisso
	Esther Duarte-Oller
	Josep Medina-Casanovas
	Fran Valenzuela-Pascual
**Additional Information:**	
**Question**	**Response**
**Financial Disclosure**<br><br>Enter a financial disclosure statement that describes the sources of funding for the	The funders had no role in study design, data collection and analysis, decision to publish, or preparation of the manuscript.

Powered by Editorial Manager® and ProduXion Manager® from Aries Systems Corporation

work included in this submission. Review the submission guidelines for detailed requirements. View published research articles from *PLOS ONE* for specific examples.<br><br>This statement is required for submission and **will appear in the published article** if the submission is accepted. Please make sure it is accurate.<br><br>**Unfunded studies**<br>Enter: *The author(s) received no specific funding for this work.*<br><br>**Funded studies**<br>Enter a statement with the following details:<br>• Initials of the authors who received each award<br>• Grant numbers awarded to each author<br>• The full name of each funder<br>• URL of each funder website<br>• Did the sponsors or funders play any role in the study design, data collection and analysis, decision to publish, or preparation of the manuscript?<br>• **NO** - Include this sentence at the end of your statement: *The funders had no role in study design, data collection and analysis, decision to publish, or preparation of the manuscript.*<br>• **YES** - Specify the role(s) played.<br><br>* typeset	
**Competing Interests**<br><br>Use the instructions below to enter a competing interest statement for this submission. On behalf of all authors, disclose any competing interests that could be perceived to bias this work—acknowledging all financial support and any other relevant financial or non-financial competing interests.<br><br>This statement is required for submission and **will appear in the published article** if the submission is accepted. Please make sure it is accurate and that any funding sources listed in your Funding Information	The authors have declared that no competing interests exist.

later in the submission form are also declared in your Financial Disclosure statement.<br><br>View published research articles from *PLOS ONE* for specific examples.<br><br>**NO authors have competing interests**<br><br>Enter: *The authors have declared that no competing interests exist.*<br><br>**Authors with competing interests**<br><br>Enter competing interest details beginning with this statement:<br><br>*I have read the journal's policy and the authors of this manuscript have the following competing interests: [insert competing interests here]*<br><br>* typeset	
**Ethics Statement**<br><br>Enter an ethics statement for this submission. This statement is required if the study involved:<br><br>• Human participants<br>• Human specimens or tissue<br>• Vertebrate animals or cephalopods<br>• Vertebrate embryos or tissues<br>• Field research<br><br>Write "N/A" if the submission does not require an ethics statement.<br><br>General guidance is provided below. Consult the submission guidelines for detailed instructions. **Make sure that all information entered here is included in the Methods section of the manuscript.**	N/A

**Format for specific study types**<br><br>**Human Subject Research (involving human participants and/or tissue)**<br>• Give the name of the institutional review board or ethics committee that approved the study<br>• Include the approval number and/or a statement indicating approval of this research<br>• Indicate the form of consent obtained (written/oral) or the reason that consent was not obtained (e.g. the data were analyzed anonymously)<br><br>**Animal Research (involving vertebrate animals, embryos or tissues)**<br>• Provide the name of the Institutional Animal Care and Use Committee (IACUC) or other relevant ethics board that reviewed the study protocol, and indicate whether they approved this research or granted a formal waiver of ethical approval<br>• Include an approval number if one was obtained<br>• If the study involved *non-human primates*, add *additional details* about animal welfare and steps taken to ameliorate suffering<br>• If anesthesia, euthanasia, or any kind of animal sacrifice is part of the study, include briefly which substances and/or methods were applied<br><br>**Field Research**<br><br>Include the following details if this study involves the collection of plant, animal, or other materials from a natural setting:<br>• Field permit number<br>• Name of the institution or relevant body that granted permission	
**Data Availability**<br><br>Authors are required to make all data underlying the findings described fully available, without restriction, and from the time of publication. PLOS allows rare exceptions to address legal and ethical concerns. See the PLOS Data Policy and FAQ for detailed information.	Yes - all data are fully available without restriction

Powered by Editorial Manager® and ProduXion Manager® from Aries Systems Corporation

A Data Availability Statement describing where the data can be found is required at submission. Your answers to this question constitute the Data Availability Statement and **will be published in the article**, if accepted.<br><br>**Important:** Stating 'data available on request from the author' is not sufficient. If your data are only available upon request, select 'No' for the first question and explain your exceptional situation in the text box.<br><br>Do the authors confirm that all data underlying the findings described in their manuscript are fully available without restriction?	
**Describe where the data may be found in full sentences. If you are copying our sample text, replace any instances of XXX with the appropriate details.**<br><br>• If the data are **held or will be held in a public repository**, include URLs, accession numbers or DOIs. If this information will only be available after acceptance, indicate this by ticking the box below. For example: *All XXX files are available from the XXX database (accession number(s) XXX, XXX.).*<br>• If the data are all contained **within the manuscript and/or Supporting Information files**, enter the following: *All relevant data are within the manuscript and its Supporting Information files.*<br>• If neither of these applies but you are able to provide **details of access elsewhere**, with or without limitations, please do so. For example:<br><br>*Data cannot be shared publicly because of [XXX]. Data are available from the XXX Institutional Data Access / Ethics Committee (contact via XXX) for researchers who meet the criteria for access to confidential data.*<br><br>*The data underlying the results presented in the study are available from (include the name of the third party*	All relevant data are within the manuscript and its Supporting Information files.

and contact information or URL).<br>• This text is appropriate if the data are owned by a third party and authors do not have permission to share the data.<br><br>* typeset	
Additional data availability information:	Tick here if the URLs/accession numbers/DOIs will be available only after acceptance of the manuscript for publication so that we can ensure their inclusion before publication.

Manuscript Click here to access/download;Manuscript;Final version_2.doc

Developing An Assistive Technology Usability Questionnaire for People with Neurological Diseases

Maria Masbernat-Almenara[1,2,3]; Francesc Rubi-Carnacea [1,2,3*]; Eloy Opisso [4,5,6]; Esther Duarte-Oller [7, 8]; Josep Medina-Casanovas [4,5,6;] Fran Valenzuela-Pascual[1,2,3]

[1] Department of Nursing and Physiotherapy, University of Lleida, Lleida, Spain

[2] Research group of health care. IRB Lleida, Institute for Biomedical Research Dr. Pifarré Foundation, Lleida, Spain

[3] Group on Society Studies, Health, Education and Culture, University of Lleida, Spain

[4] Institut Guttmann, Neurorehabilitation Institute, Camí de Can Ruti, Badalona, Spain

[5] Universitat Autònoma de Barcelona, Plaça Cívica, Cerdanyola del Vallés, Barcelona, Spain

[6] Fundació Institut d'investigació en Ciències de la Salut Germans Trias i Pujol, Carretera Canyet, Badalona, Barcelona, Spain

[7] Physical Medicine and Rehabilitation Department, Parc de Salut Mar (Hospital del Mar, Hospital de l'Esperança). Barcelona, Catalonia, Spain

[8] Rehabilitation Research Group, Hospital del Mar Medical Research Institute (IMIM). Barcelona, Catalonia, Spain.

*Corresponding Author:

Francesc Rubí Carnacea, Ph.D, Email: francesc.rubi@udl.cat

Abstract

Purpose. This study describes the development of a questionnaire for assessing the usability of assistive technologies accessible to people with neurological diseases. *Methods.* A Delphi study was conducted to identify relevant items for the questionnaire. After that, the content validity was addressed to identify the essential items. Once the questionnaire was designed following the results of Delphi study and content validity, the reliability and validity, and the rasch model of the questionnaire were examined. *Results*. Two rounds of the Delphi study were carried out. A total 73 participants (42 experts and 31 users) participated in round 1 and 59 people (27 experts and 32 users) in round 2. A total of 53 and 29 items were identified in rounds 1 and 2, respectively. In the content validity we found nine items above the threshold of 0.58. Finally, 10 items were included in the questionnaire. 51 participants participate in the reliability and validity of the questionnaire. Internal consistency reliability of the questionnaire analyzed by Cronbach's Alpha was $\alpha=0,895$. There was moderate to considerable concordance among our questionnaire items test-retest in the Kappa coefficient and a strong association between test-retest in the Spearman's coefficient $\rho=0.818$ ($p<0,001$). The intraclass correlation coefficient was 0,869 with 95% of confidence interval (0,781;0,923). There was a strong correlation of the total scores between the new questionnaire and other validate questionnaire analyzed with Spearman's coefficient $\rho=0.756$ ($p<0,001$). The 10 items demonstrated a satisfactory fit to the Rasch model. *Conclusions*. The present study suggested that the new questionnaire is a reliable 10-item usability questionnaire that allows subjective and quick assessment of the usability of assistive technologies by people with neurological diseases.

Key words: Nervous System Diseases, Neurological Rehabilitation, Questionnaire Design, Self-Help Devices, User-centered Design

Introduction

Neurological disorders are among the most important causes of disability (247–308 million) and death (8·8–9·4 million), worldwide. [1]In addition, the burden of neurological disorders in public health has increased substantially in the last 25 years because of expanding population numbers, aging, and an increase in survivor rates from stroke and other neurological disorders.[2] These survivors require intensive rehabilitation to reduce the sequelae of the disorders, increase their quality of life, and improve their autonomy in activities of daily living. In most cases, some assistive technology is needed.

Recent advances in the field of technology and rehabilitation have led to the development of new tools to asses' people with disabilities and to improve their functionality and their autonomy in their daily life.[3] It is known that a good acceptance of the assistive technology can improve the quality of life and social inclusion of these patients[4]. However, not all products achieve the goal for which they were designed since they often do not take into account the real needs of users.[5] It is therefore increasingly necessary to involve end-users, from the beginning of the development of new products.[6] A product can only be considered successful if it is used, and currently, more than 50% of users abandon their new products because these are not sufficiently usable.[7,8] For this reason, usability is becoming more important in the field of engineering and rehabilitation. The design of a product should not involve only multidisciplinary theoretical foundations, but user experience should be considered throughout the design process to ensure quality, improve usability, and increase product acceptability.[5,9]

Assistive technology term includes any item, piece of equipment, whether acquired or off the shelf, modified or customized, that is used to increase, maintain, or improve functional capacities of individuals with disabilities[10].

There is a lack of evidence-based procedures for assistive technologies selection [11–13] , however the clinical outcome assessment is important, both in clinical practice

and in research because among others, it improves evidence, and it provides considerable feedback to both healthcare profesionals and patients enhancing their empowerment related to their quality of life[14].

Nowadays, a high proportion of existing usability questionnaires were developed to evaluate software usability and web accessibility[15] and not assistive technologies [16]. Some questionnaires[17–19] measure the psychosocial impact of quality of life by using assistive tehcnologies from the point of view of people with disabilities. Those are interesting questionnaires; however, they do not evaluate the assistive technologies per se. There are other questionnaires developed exclusively to assess wheelchairs[20] and only a few depeveloped specificaly for assistive technologies. However, developing a new prototype of assistive technology [9] we found that people with neurological diseases have limitations in the accessibility in the existing usability questionnaires in answering technical development questions, for example the Quebec User Evaluation of Satisfaction with Assistive Technology (QUEST 2.0) [21] contains technical items (weight, dimensions of the product, services delivery, repairs, and services of the device). In adittion, existing questionnaires are too extensive for them or do not address all the essential items for assessing usability and user satisfaction.[16,22] It is known that longer questionnaires incur high costs in data collection and reduce the number of answers due to the time answering and the quality of the information gathered due to the fatigue.[23,24]

The objective of this study was to develop a short questionnaire focused only on the usability of assistive technology products and user satisfaction and it should be accessible and easy to understand to people with neurological diseases.

Materials and methods

Questionnaire Design

Delphi study

To construct an easier to use questionnaire we performed a Delphi study,[25] as it is a prospective approach that seeks to derive a consensus from a group of experts, based on the analysis and reflection of a defined problem. A google form questionnaire was sent via

email to the health care professionals from the private neurorehabilitation hospital of acute neurological patients (Institut Guttmann, Spain) and public hospital of acute stroke patients (Hospital de l'Esperança, Spain).

The questionnaire contains six questions: three were open-questions related to assistive technologies, and three were multiple-choice questions related to how a questionnaire should be to be easy and quick to answer. The 3 open-questions were: (1) List some requirements or characteristics that are important to how the assistive products should be or how they should be manufactured. (2) Would you change anything about the existing assistive products? (3) What kind of assistive products would you like to have? (for users)/ in your workplace? (experts). The three multiple-choice questions related to the questionnaires were: (1) the time required for you to answer a questionnaire, (2) which questionnaire format the user preferred, and (3) how many questions you would like to answer.

Content Validity Ratio

Once the Delphi study finished and the items were obtained, they were evaluated using the content validity ratio (CVR).[26,27] The content validity addresses the degree to which items of an instrument sufficiently represent the content domain and answers the question that to what extent the selected sample in an instrument or instrument items is a comprehensive sample of the content. [27] Content validity ratio varies between 1 and -1. Higher scores indicate greater agreement by panel members on the need for an item in an instrument. The formula of content validity ratio is CVR= (Ne - N/2)/(N/2), in which the Ne is the number of panelists indicating "essential" and N is the total number of panelists.[27] The numeric value of content validity ratio is determined by Lawshe Table.[26]

Questionnaire design

The questions were written based on the qualitative data obtained in the Delphi study and the items obtained in the CVR. Subsequently, the users (people with neurological diseases) read the questionnaire to assess their understanding of the questions and if their answers could be represented with the proposed response values.

Questionnaire reliability and validity

The reliability and validity of the new questionnaire will be addressed.

Sample size. The recommended sample size for similar studies has been established as 50 subjects.[28,29] The inclusion criteria for participants were adults (≥18 years old) with neurological diseases that have been using some assistive technology for the past month and Pfeiffer SPMSQ test [30] translated into Spanish[31] punctuation was between 0 and 2 mistakes.

Assistive technology analyzed: It is difficult to analyze the same product because neurological patients have personalized assistive technologies adapted to their disability. For that reason, all the participants could choose which product addressed, only with the condition that they had used it for at least one month.

Data analysis of Reliability

The reliability and validity of the new questionnaire and all the data were analyzed using the SPSS Statistics 27 program.

The internal consistency reliability of the new questionnaire was analyzed using the Cronbach's Alpha.[32] The value of the coefficients were interpreted as follows: unacceptable ($0.5 \geq \alpha$), poor ($0.6 \geq \alpha \geq 0.5$), questionable ($0.7 \geq \alpha \geq 0.6$), acceptable ($0.8 \geq \alpha \geq 0.7$). good ($0.9 \geq \alpha \geq 0.8$), and excellent $\alpha \geq 0.9$).[32]

Test-retest. Reliability can be assessed with a test-retest comparison. This method evaluates the stability of the results at two different points in time by means of a stability coefficient. To perform the test-retest, the same questionnaire was administered on two occasions, separated by a certain period, to the same subjects and under the same conditions. According to several authors,[33] an interval of between 2 days and 2 weeks between the test-retest interviews is recommended. Accordingly, the interval between the two tests was set to 15 days. In order to know the concordance between the test and the retest, data was analyzed by weighted quadratic Kappa coefficient. [34] The value of the coefficients were interpreted as follows: poor (< 0.00), weak (0.00–0.20), good (0.20–0.40), moderate (0.41–0.60), considerable (0.61–0.80), and almost perfect (0.81–1.00).[34]

Intraclass correlation coefficient (ICC) with 95% of confidence interval (CI) was measured. ICC indicates the degree to which the participants maintained their opinion during the test-retest.[35] [illegible] value of the coefficients were interpreted as follows: poor reliability (< 0.5), moderate reliability (0.5–0.75), good reliability (0.75–0.9), excellent reliability (> 0.90).[35]

Normality of the data was tested using the Kolmogorov-Smirnov test and the repeatability was determined by the Spearman's correlation coefficient[36] between the test and the retest.

Data Analysis of validity

Construct validity. To know the relationship between the new questionnaire and another related questionnaire, QUEST 2.0[21] was chosen. It is the shortest worldwide used questionnaire, and it has been translated and validated in several languages.Only eight items of the QUEST 2.0 was administered to users in Spanish[37] because the other 4 items are related to services. Also, in this study, data was tested using the Kolmogorov-Smirnov test, and it was analyzed using the Spearman's correlation coefficient [36] .

Measurement of other parameters. The time was measured while the participants answer the questionnaire, in order to obtain the average time of all the participants.

Rasch Model

In order to analize deepely the behavior of the different items, the Rasch model was carried out. This model provide an idea of the internal consistency of the scale due to match the item difficulty with the person's ability[38]. The values of infit and outfit mean squares (MNSQ) of 1 indicate a perfect fit between the data and the model, values between 0.5 and 1.5 indicate an acceptable fit, and values greater than 2, indicate a severe mismatch[38].

Ethical Approval

The Institut Guttmann Neurorehabilitation Hospital Ethics Committee approved this study. In addition, this research was conducted following the Declaration of Helsinki's ethical principles. All participants participated voluntarily, and they signed an informed

consent form. Their personal data were archived following the Spanish Organic Law 3/2018, of December 5, on the Protection of Personal Data and guarantee of digital rights.

The study was developed following the COSMIN guidelines[39].

Results

Study Delphi

Two rounds of a Delphi study were needed to obtain all the items of the new questionnaire. A total of 73 participants (42 experts and 31 users) were involved in round 1 (see Table I). From round 1, 53 different items and qualitative information about questionnaires were derived.

In round two, 59 people (27 experts and 32 users) (see table 1). From this round, 15 items and qualitative data about the scale were obtained. The items were: “effectiveness”, “comfort”, “adaptability”, “easy to put on/off”, “safe”, “lightweight”, “functionality”, “ergonomic”, “economical”, “affordable”, “easy to use”, “feedback”, “stimulating,” “monitored” and “movement facilitator. Due to “economical” and “affordable” have the same meaning, both were considered as a single item. Additionally, in this round and following the usability premises, the items “esthetics” and “easy to remember how to use it” were added. Finally, 16 items were analyzed using the content validity ratio.

Table 1: Demographic Characteristics of participants of the two rounds in the Delphi Study.

**EXPERT PANEL**	**Round 1 n=42**		**Round 2 n= 27***	
**Demographic Characteristics**	**Number**	**Percentage**	**Number**	**Percentage**
**Age**				
Between 18 and 25 years	n= 3	7%	n=1	4%
Between 26 and 35 years	n=17	41%	n=11	41%
Between 36 and 45 years	n=11	26%	n=8	29%
Between 46 and 65 years	n= 11	26%	n=7	26%
More than 65 years	n= 0	0%	n=0	0%

**Sex**				
Female	N=30	71%	19	70%
Male	N=12	29%	8	30%
**Profession**				
Physician	n=9	21%	6	22%
Physiotherapist	n=14	33%	9	33%
Occupational Therapist	n=13	31%	8	30%
**Other	n=6	15%	4	15%
**Expert in Neurorehabilitation**				
SI	39	93%	27	100%
**USER PANEL**	**Round 1 n= 31**		**Round 2 n= 32 *****	
**Demographic Characteristics**	**Number**	**Percentage**	**Number**	**Percentage**
**Age**				
Between 18 and 25 years	n= 4	13%	n=1	3%
Between 26 and 35 years	n=6	19%	n=9	28%
Between 36 and 45 years	n=5	16%	n=3	10%
Between 46 and 65 years	n= 13	42%	n=16	50%
More than 65 years	n= 3	10%	n=3	9%
**Sex**				
Female	n=17	45%	n=16	50%
Male	n=14	55%	n=16	50%
**Pathology**				
Spinal Cord Injury	n=13	42%	n=7	22%
Traumatic Brain injury	n=5	16%	n=1	3%
Stroke	n=8	26%	n=13	41%
Other	n=5	16%	n=11	34%
**Assistive Technologies**				
Wheelchair	n= 23	53%	n=19	36%
Canes	n= 8	18%	n=12	23%
Orthesis	n=8	19%	n=15	28%
Other	n=2	5%	n=6	11%
Nothing	n=2	5%	n=1	2%

*In the second round, 36% did not answer the questionnaire. ** Other are, for example, orthopaedic professionals and engineers. *** Users are different from the round 1.

Content Validity Ratio (CVR)

34 experts from the private neurorehabilitation hospital of acute neurological patients and the public hospital of acute stroke patients were selected to participate to evaluate the

items obtained from the round two. 70% of the participants have more than 10 years of expertise in neurorehabilitation and all of them used to work with assistive technologies experts (see table 2).

Table 2: Demographic Characteristic of participants in Content Validity

	**Content Validity n=34**	
**Demographic Characteristics**	**Number**	**Percentage**
**Age**		
Between 26 and 35 years	n=8	24%
Between 36 and 45 years	n=12	35%
Between 46 and 65 years	n= 14	41%
**Sex**		
Female	N=21	62%
Male	N=13	38%
**Profession**		
Physician	n= 11	32%
Physiotherapist	n=14	41%
Occupational Therapist	n=7	21%
*Other	n=2	6%
****Expert in Neurorehabilitation**		
Between 1 and 5 years	n=5	15%
Between 5 and 10 years	n=5	15%
Between 10 and 20 years	n=12	35%
More than 20 years	n=12	35%

* Other are, for example, orthopaedic professionals and engineers. *All the participants used to work with people with neurological diseases.

Participants had to choose which items were *essential*, *useful but not essential,* and *not essential* when evaluating the items. Table 3 showed the results, nine of the sixteen items exceeded the threshold of 0.58. In addition, the experts were in consensus to accept the item “comfortable,” because its threshold was 0.53.

Table 3. Content Validity Ratio’ results.

**Items**	**ne**	**N**	**CVR***

1. Effectiveness	33	34	0,97
2. Comfortable	18	34	0,53
3.Adaptability	21	34	0,62
4. Easy to put on/off	20	34	0,59
5. Safe	32	34	0,94
6. Lightweight	12	34	0,35
7. Functionality	31	34	0,91
8. Ergonomic	22	34	0,65
9.Economical/ Affordable	13	34	0,38
10. Easy to use	24	34	0,71
11. Esthetics	4	34	0,12
12. Easy to remember how to use it	20	34	0,59
13. Feedback	16	34	0,47
14. Stimulating	14	34	0,41
15. Monitored	10	34	0,29
16. Movement Facilitator	21	34	0,62

*CVR (content validity ratio) = (Ne-N/2) / (N/2) with 34 people at the expert panel (N=34), the items with the CVR bigger than 0.58 (in bold) will remain at the instrument and the rest will be eliminated.

The experts considered “functional” and “movement facilitator” as one word due to the similarity of their meanings. The item “satisfaction” was added to the questionnaire to ascertain the end-user opinion of the product. Therefore, 10 items were selected to create the questionnaire (see Table 4).

Table 4. Questions and the items that are involved in each question.

**Questions**		**Items**
1	I believe that _____ can help me improve my functional independence	Effectiveness
2	I feel comfortable wearing/using ____	Comfortable
3	_____ adapts to my characteristics and needs	Adaptability
4	Donning/Doffing …………….. is quick and easy for me.	Easy to put on / off
5	I feel safe using/wearing _____ / _____ is safe in its use	Safe
6	_____ allows me to achieve my goal/ allows me to perform a movement/action that I could not do before	Functionality/ movement facilitator
7	_____ adapts to my daily live needs	Ergonomics
8	In general, _____ is easy to use	Easy to use
9	Information and instructions of use______ are easy to understand and easy to remember	Easy to remember how to use it
10	Overall, I am satisfied with______	Satisfaction

Questionnaire design

Following the qualitative information of the participants from the Delphi study, the questionnaire was formulated in an understandable language, and the length of the questionnaire was as short as possible. When necessary, the users could fill the blank space with the name of the product that was being evaluated. The 6-point Likert scale was chosen since it forces the respondent to decide positively or negatively according to the item in question[40]. Numeric panel from 0 to 5 points was used, and each number was associated with a box using the colors of the traffic light to facilitate the choice. Furthermore, faces with expressions were added to facilitate the answers according to the patients with neurological diseases responses from the Delphi study.

The statements of the questions are in first person in order to facilitate users answers and usability experiences of the product subjectively.

Once the questionnaire was finished, ten users analysed it to know if all the requirements were met. During this process, some wording modifications were made.

The new questionnaire was named "Assistive Technology Usability Questionnaire for people with Neurological diseases" (NATU Quest) and included 10 questions. It should be administered at the end of a usability test. The final version of the questionnaire, questionnaire score and interpretation are available in supplementary material.

administered at the end of a usability test

Reliability and Validity

Sample description

A total of 51 patients consecutive recruited from the Intitut Guttmann Hospital voluntary agreed to participate in the study. The demographic characteristics of the participants are summarized in Table 5. Fifty-three percent of the participants answered the questionnaire through an interview, due to physical limitations, and the rest (47%) answered the questionnaire by themselves. First, the participants answered the new questionnaire and the QUEST 2.0. On average, the participants completed the new questionnaire within 102.40 seconds in the first round and within 82.08 seconds in the second round. The

QUEST 2.0 was administered once before the NATU Quest and the participants needed an average of 74 seconds to complete it. Each participant scored an assistive technology that they had being used in the last 3 months. All the patients answered all the items.

Table 5: Demographic characteristics of the 51 participants in the reliability and validity of NATU Quest.

**Demographic Characteristics**	**Mean /Number**	**Percentage**
Age, range 16-72 years	Mean = 48	
**Sex**		
Female	n=20	39%
Male	n= 31	61%
**Pathology**		
Spinal cord Injury	n=22	43%
Traumatic Brain injury	n=3	6%
Stroke	n=16	31%
Brain injury	n=4	8%
Other neurological diseases	n=6	12%
**Education**		
Secondary	n=13	25%
High School Diploma	n=1	2%
Certificate of Professional Standards	n=15	29%
Higher Level Education Cycle (CFGS)	n=7	14%
University Degree	n=15	30%
**Questionnaire form**		
Self-administration	n=24	53%
Interview	n=27	47%
**Assistive Technologies**		
Wheelchair	n=29	
Walking stick	n=8	
Walker	n=3	
Splint	n=7	
Treadmill	n=5	

**Pfeiffer Test**		
Normal (0-2 mistakes)	n=51	100%
Low cognitive impairment (3-4)	n=0	
Mild cognitive impairment (5-7)	n=0	
Severe cognitive impairment (8-10)	n=0	

Reliability Results

The internal consistency reliability of the NATU Quest was analyzed using the Cronbach's Alpha[32] (α=0,895). This result can be interpreted as good reliability.

Reliability through test-retest. To assess the reliability of the NATU Quest, a retest was performed 15 days after answering the two questionnaires for the first time. Table 6 shows the weighted quadratic Kappa coefficient and Spearman's coefficient results of the NATU Quest. The results showed a moderate to considerable concordance between NATU Quest items test-retest in the Kappa coefficient because all the results were above 0,50. The results also showed a strong association between test-retest in the Spearman's coefficient (ρ = 0.818), significant with p value < 0.0001. The results of the ICC showed good reliability (ICC = 0,869; CI 95% 0,781 to 0,923).

Table 6: NATU Quest reliability of the test-retest

**NATU Quest**				
**Items**		**Weighted Kappa**	**95% Confidence Interval**	
			**Lower Limit**	**Upper Limit**
1	Effectiveness	0,727	0,535	0,918
2	Comfortable	0,733	0,645	0,821
3	Adaptability	0,585	0,434	0,735
4	Easy to put on/off	0,631	0,399	0,863
5	Safe	0,551	0,206	0,895
6	Functionality	0,685	0,541	0,828
7	Ergonomics	0,673	0,507	0,840
8	Easy to use	0,544	0.332	0,755
9	Easy to remember how to use it	0,624	0,298	0,950
10	Satisfaction	0,714	0,557	0,871

**Spearman**	0,818**	

** The correlation is significant $p < 0.0001$ (bilateral).

Construct validity

The correlation of the total scores between NATU Quest and QUEST 2.0 analyzed with the Spearman's coefficient was strong with $\rho = 0.756$ significant, with p value < 0.0001.

Rasch Model Results

All 10 items demonstrated a satisfactory fit to the Rasch model, which could be considered productive for measurement (MNSQ infit between 0.64 and 1.43; MNSQ outfit between 0.52 and 1.49).

Discussion

There is a need for a short and easy questionaires to properly assess usability of assitive techologies in people with neurological diseases.

The items included in the questionnaire, the format of the questionnaire and the answer form were derived through two rounds of a Delphi study[25] based on the opinion of 69 experts (neurorehabilitation professionals, such as medical doctors and physiotherapists) and 63 users (people with neurological diseases). Finally, using a content validity ratio, we narrowed items down to 10 essential usability items. Some of the items also appear in other usability questionnaires such as "effectiveness" and "adaptability" are included in the PIADS,[18] while "comfort," "easy to use," "safe" "easy to put on/off," and "satisfaction" appears in the PIADS,[18] Usability Scale for Assistive Technology for Wheeled Mobility (USAT-WM),[20] and QUEST 2.0.[21] Items such as "ergonomic" and "functionality" appear in the PIADS[18] and USAT-WM.[20] Only the item "easy to remember how to use it" does not appear in any questionnaire but is an usability attribute[13].

Once the questionare form was designed, a total of 51 end-users with neurological diseases participated in the questionnaire validity and reliablity. Results suggested that NATU Quest has a good reliability and validity and it also fits in the rasch model.

In contrast with other questionnaires, the NATU Quest was developed taking into account the professionals and people with neurological diseases' opinions. Other relevant aspects of this study are the heterogeneity of the included sample, the wide range of neurological diseases and the inclusion of different assistive technologies.

In this study, we developed a usability scale to analyze assistive technologies for people with neurological diseases; however, the study had some limitations: (1) Selection bias of the participants since the majority of them were from the same province. (2) Although all the users who participated in the validation had a neurological disease, the authors chose the Pfeiffer test to assess the cognitive problems because it is short and quick to answer. However, Pfeiffer Test does not accurately assess all possible cognitive deficits, and it is not sensitive enough to detect low or mild cognitive deficits. (3) Due to the people with neurological diseases conditions, different assistive technologies were analyzed. It would be very interesting to be able to perform another validation with the same product for all users, maybe will be a good option in the developing of a new product. (4) Finally, the test-retest reliability was only compared to the QUEST 2.0; because we considered that adding other tests for comparison in the same study would have placed a burden on the users.

For future work, it would be interesting to measure test-retest reliability with another usability questionnaire. It would also be interesting to verify the new questionnaire external validity with other population groups, such as older people. Finally, the items should be reviewed after a few years to determine if they remain sufficiently sensitive for assessing rapidly evolving assistive technologies. Also, should be interesting to translate the new questionnaire to other languages.

Conclusion

The present study suggested that the NATU is a reliable 10-item usability questionnaire that allows subjective and quick assessment of the usability of assistive technologies. This questionnaire aims to be accessible to people with neurological diseases and it reflects the level of acceptance and satisfaction that a patient has with the product that is using. In addition, the NATU Quest can also be useful for evaluating products in development through user-centered design, since the patient can state an opinion about the product

during its development, which will facilitate the development of products for a better fit for patients' needs.

Conflicts of interest

The authors certify that there is no conflict of interest to declare.

Funding

The author(s) received no specific funding for this work.

Supporting information

Natu Questionnaire

Acknowledgments

The authors are grateful to all the professionals and participants involved in this study for their contribution.

Author Contributions

Idea and the project design: Maria Masbernat-Almenara; Eloy Opisso, Esther Duarte-Oller and Josep Medina-Casanovas.

Data collection: Maria Masbernat-Almenara

Data analysis: Maria Masbernat-Almenara; Eloy Opisso, Francesc Rubí-Carnacea, Fran Valenzuela-Pascual provided data analysis.

Participants, facilities, and equipment: Josep Medina-Casanovas and Esther Duarte-Oller

Institutional liaisons and consultation: Francesc Rubí-Carnacea and Fran Valenzuela-Pascual

All authors provided writing and revision. All authors read and approved the final version of the manuscript.

REFERENCES

1. Feigin VL, Nichols E, Alam T, Bannick MS, Beghi E, Blake N, et al. Global, regional, and national burden of neurological disorders, 1990–2016: a systematic analysis for the Global Burden of Disease Study 2016. Lancet Neurol. 2019;18: 459–480. doi:10.1016/S1474-4422(18)30499-X
2. Feigin VL, Krishnamurthi R V., Theadom AM, Abajobir AA, Mishra SR, Ahmed MB, et al. Global, regional, and national burden of neurological disorders during 1990–2015: a systematic analysis for the Global Burden of Disease Study 2015. Lancet Neurol. 2017;16: 877–897. doi:10.1016/S1474-4422(17)30299-5
3. De Mello Monteiro CB, Dawes H, Mayo N, Collett J, Magalhaes FH. Assistive Technology Innovations in Neurological Conditions. Biomed Res Int. 2021;2021. doi:10.1155/2021/6846120
4. Baldassin V, Shimizu HE, Fachin-Martins E. Computer assistive technology and associations with quality of life for individuals with spinal cord injury: a systematic review. Qual Life Res. 2018;27: 597–607. doi:10.1007/S11136-018-1804-9
5. Elnady A, Ben Mortenson W, Menon C. Perceptions of existing wearable robotic devices for upper extremity and suggestions for their development: Findings from therapists and people with stroke. J Med Internet Res. 2018;20: e12. doi:10.2196/rehab.9535
6. Rowland JL, Malone LA, Fidopiastis CM, Padalabalanarayanan S, Thirumalai M, Rimmer JH. Perspectives on Active Video Gaming as a New Frontier in Accessible Physical Activity for Youth With Physical Disabilities. Phys Ther. 2016;96: 521–532. doi:10.2522/PTJ.20140258
7. Sugawara AT, Ramos VD, Alfieri FM, Battistella LR. Abandonment of assistive products: assessing abandonment levels and factors that impact on it. Disabil Rehabil Assist Technol. 2018;13: 716–723. doi:10.1080/17483107.2018.1425748
8. Federici S, Meloni F, Borsci S. The abandonment of assistive technology in Italy: A survey of National Health Service users. Eur J Phys Rehabil Med. 2016;52: 516–526.
9. Almenara M, Cempini M, Gómez C, Cortese M, Martín C, Medina J, et al. Usability test of a hand exoskeleton for activities of daily living: an example of user-centered design. Disabil Rehabil Assist Technol. 2017;12: 84–96. doi:10.3109/17483107.2015.1079653

10. McSweeney-Feld MH. Assistive Technology and Older Adults in Disasters: Implications for Emergency Management. Disaster Med Public Health Prep. 2017;11: 135–139. doi:10.1017/DMP.2016.160
11. Friederich A, Bernd T, De Witte L. Methods for the selection of assistive technology in neurological rehabilitation practice. Scand J Occup Ther. 2010;17: 308–318. doi:10.3109/11038120903377082
12. Bernd T, Van Der Pijl D, De Witte LP. Existing models and instruments for the selection of assistive technology in rehabilitation practice. Scand J Occup Ther. 2009;16: 146–158. doi:10.1080/11038120802449362
13. Carneiro L, Rebelo F, Filgueiras E, Noriega P. Usability and User Experience of Technical Aids for People with Disabilities? A Preliminary Study with a Wheelchair. Procedia Manuf. 2015;3: 6068–6074. doi:10.1016/J.PROMFG.2015.07.736
14. Grimm B, Blom A, Jahr H, Rosenbaum D. New Tools and Technologies for Clinical Outcome assessment. J Orthop Transl. 2016;7: 70. doi:10.1016/J.JOT.2016.06.020
15. Brooke john. SUS: A “Quick and Dirty” Usability Scale. 1996; 207–212. doi:10.1201/9781498710411-35
16. Koumpouros Y. A Systematic Review on Existing Measures for the Subjective Assessment of Rehabilitation and Assistive Robot Devices. Journal of Healthcare Engineering. Hindawi Limited; 2016. doi:10.1155/2016/1048964
17. Demers L, Monette M, Descent M, Jutai J, Wolfson C. The psychosocial impact of assistive devices scale (PIADS): Translation and preliminary psychometric evaluation of a Canadian-French version. Qual Life Res. 2002;11: 583–592. doi:10.1023/A:1016397412708
18. Jutai J, Day H. Psychosocial Impact of Assistive Devices Scale (PIADS). Technol Disabil. 2002;14: 107–111. doi:10.3233/tad-2002-14305
19. Scherer MJ, Cushman LA. Measuring subjective quality of life following spinal cord injury: A validation study of the assistive technology device predisposition assessment. Disabil Rehabil. 2001;23: 387–393. doi:10.1080/09638280010006665
20. Arthanat S, Nochajski SM, Lenker JA, Bauer SM, Wu YWB. Measuring usability of assistive Technology from a multicontextual perspective: The case of power wheelchairs. Am J Occup Ther. 2009;63: 751–764. doi:10.5014/ajot.63.6.751

21. Demers L, Weiss-Lambrou R, Ska B. The Quebec User Evaluation of Satisfaction with Assistive Technology (QUEST 2.0): An overview and recent progress. Technol Disabil. 2002;14: 101–105. doi:10.3233/tad-2002-14304
22. Almenara Masbernat M, Medina Casanovas J, Duarte Oller E, Selva O'Callaghan A, Universitat Autònoma de Barcelona. Departament de Medicina. Modelo teórico-práctico para la implementación del diseño centrado en el usuario en el desarrollo, la validación y la aceptación de los productos de apoyo para personas con enfermedades de origen neurológico. 2018.
23. Lavrakas P. Encyclopedia of Survey Research Methods. Encyclopedia of Survey Research Methods. Sage Publications, Inc.; 2012. doi:10.4135/9781412963947
24. Choi YM, Sprigle SH. Approaches for evaluating the usability of assistive technology product prototypes. Assist Technol. 2011;23: 36–41. doi:10.1080/10400435.2010.541407
25. Landeta J. Current validity of the Delphi method in social sciences. Technol Forecast Soc Change. 2006;73: 467–482. doi:10.1016/j.techfore.2005.09.002
26. Tristán-López A. Modificación al modelo de Lawshe para el dictamen cuantitativo de la validez de contenido de un instrumento objetivo. Av en medición. 2008;6: 37–48.
27. Zamanzadeh V, Ghahramanian A, Rassouli M, Abbaszadeh A, Alavi-Majd H, Nikanfar A-R. Design and Implementation Content Validity Study: Development of an instrument for measuring Patient-Centered Communication. J Caring Sci. 2015;4: 165–178. doi:10.15171/jcs.2015.017
28. Jackson JL, Chamberlin J, Kroenke K. Predictors of patient satisfaction. Soc Sci Med. 2001;52: 609–620. doi:10.1016/S0277-9536(00)00164-7
29. Bowling A, Rowe G, Lambert N, Waddington M, Mahtani KR, Kenten C, et al. The measurement of patients' expectations for health care: A review and psychometric testing of a measure of patients' expectations. Health Technology Assessment. 2012. pp. 1–532. doi:10.3310/hta16300
30. Pfeiffer E. A Short Portable Mental Status Questionnaire for the Assessment of Organic Brain Deficit in Elderly Patients. J Am Geriatr Soc. 1975;23: 433–441. doi:10.1111/j.1532-5415.1975.tb00927.x
31. Martínez de la Iglesia J, Dueñas Herrero R, Onís Vilches MC, Aguado Taberné C, Albert Colomer C, Luque Luque R. [Spanish language adaptation and validation of

the Pfeiffer’s questionnaire (SPMSQ) to detect cognitive deterioration in people over 65 years of age]. Med Clin (Barc). 2001;117: 129–34. Available: http://www.ncbi.nlm.nih.gov/pubmed/11472684

32. George D, Mallery P. SPSS for Windows step by step : a simple guide and reference, 17.0 update. 4th ed. Boston: Allyn & Bacon, editor. 2003.

33. Streiner DL, Norman GR, Cairney J. Oxford Medicine Online Health Measurement Scales: A practical guide to their development and use (5 ed.). Chapter: Reliability. 2019. doi:10.1093/med/9780199685219.001.0001

34. Fleiss JL, Cohen J. The Equivalence of Weighted Kappa and the Intraclass Correlation Coefficient as Measures of Reliability: http://dx.doi.org/101177/001316447303300309. 2016;33: 613–619. doi:10.1177/001316447303300309

35. Koo TK, Li MY. A Guideline of Selecting and Reporting Intraclass Correlation Coefficients for Reliability Research. J Chiropr Med. 2016;15: 155. doi:10.1016/J.JCM.2016.02.012

36. Mukaka M. A guide to appropriate use of Correlation coefficient in medical research. Malawi Med J. 2012;24: 69. Available: /pmc/articles/PMC3576830/

37. Mora Barrera CA. Validación de la versión en español de la evaluación Quebec de usuarios con tecnologia de asistencia (QUEST 2.0). Universidad Nacional de Colombia. 2010.

38. Boone WJ. Rasch Analysis for Instrument Development: Why, When, and How? CBE Life Sci Educ. 2016;15. doi:10.1187/CBE.16-04-0148

39. Terwee CB, Prinsen CAC, Chiarotto A, Westerman MJ, Patrick DL, Alonso J, et al. COSMIN methodology for evaluating the content validity of patient-reported outcome measures: a Delphi study. Qual Life Res. 2018;27: 1159–1170. doi:10.1007/s11136-018-1829-0

40. Abdul. Quality of Psychology Test Between Likert Scale 5 and 6 Points. J Soc Sci. 2010;6: 399–403. doi:10.3844/jssp.2010.399.403

Click here to access/download
Supporting Information
NATU Quest Users Questionnaire - Supporting Information.docx
