# Supplementary material for: Developing an assistive technology usability questionnaire for people with neurological diseases
Source: PLoS One. 2023 Jan 31;18(1):e0281197. doi: 10.1371/journal.pone.0281197 (PMC9888697; doi:10.1371/journal.pone.0281197)
Supplement: Attachment — Submitted filename: PONE-D-22-13676_R1 HL.pdf [file pone.0281197.s006.pdf]

PLOS ONE

Developing An Assistive Technology Usability Questionnaire for People with Neurological Diseases

--Manuscript Draft--

**Manuscript Number:**	PONE-D-22-13676R1
**Article Type:**	Research Article
**Full Title:**	Developing An Assistive Technology Usability Questionnaire for People with Neurological Diseases
**Short Title:**	Assistive Technology Usability Questionnaire for people with Neurological Diseases
**Corresponding Author:**	Francesc Rubí-Carnacea<br>University of Lleida: Universitat de Lleida<br>Lleida, Lleida SPAIN
**Keywords:**	Nervous System Diseases, Neurological Rehabilitation, Questionnaire Design, Self-Help Devices, User-centered Design
**Abstract:**	Purpose. This study describes the development of a questionnaire for assessing the usability of assistive technologies accessible to people with neurological diseases. Methods. A Delphi study was conducted to identify relevant items for the questionnaire. After that, the content validity was addressed to identify the essential items. Once the questionnaire was designed following the results of the Delphi study and content validity, the reliability, validity, and the Rasch model of the questionnaire were examined. Results. Two rounds of the Delphi study were carried out. A total of 73 participants (42 experts and 31 users) participated in round 1, and 59 people (27 experts and 32 users) in round 2. A total of 53 and 29 items were identified in rounds 1 and 2, respectively. In the content validity, we found nine items above the threshold of 0.58. Finally, ten items were included in the questionnaire. Fifty-one participants participate in the reliability and validity of the questionnaire. The internal consistency reliability of the questionnaire analyzed by Cronbach's Alpha was α=0,895. There was moderate to considerable concordance among our questionnaire items test-retest in the Kappa coefficient and a strong association between test-retest in the Spearman's coefficient ρ=0.818 (p<0,001). The intraclass correlation coefficient was 0,869 with a 95% confidence interval (0,781;0,923). There was a strong correlation between the total scores of the new questionnaire and other validated questionnaires analyzed with Spearman's coefficient ρ=0.756 (p<0,001). The ten items demonstrated a satisfactory fit to the Rasch model. Conclusions. The present study suggested that the new questionnaire is a reliable 10-item usability questionnaire that allows subjective and quick assessment of the usability of assistive technologies by people with neurological diseases.
**Order of Authors:**	Maria Masbernat-Almenara
	Francesc Rubí-Carnacea
	Eloy Opisso
	Esther Duarte-Oller
	Josep Medina-Casanovas
	Fran Valenzuela-Pascual
**Response to Reviewers:**	REVIEWER 1<br>1. Assistive Technologies, instead of Nervous System Diseases????<br>We thank the reviewer's observation. MESH term of Assistive Technologies is Self-Help Devices.<br><br>2. functioning, instead<br>We thank the reviewer's observation. However, we want to express functionality instead of functioning.<br><br>3. This definition is very questionable! The authors have taken and written ipsis verbis

from [10] who, in turn, took it from Fed Regist. 1991;56(60):4121... 1991!!!!!!!!!!!!!!!!! It will be useful to reformulate this text and, consequently, the conceptual framework of this definition.
According to the reviewer, we have added a new definition. Please see page 4, line 76 and below.
According to the WHO, assistive technology is a general term covering the systems and services related to delivering assistive products and services.[12] Assistive products aim to maintain or improve an individual's functioning and independence, promoting their well-being.[12]
4. To use the term "person with neurological diseases" is not entirely correct. I think the authors want to develop a tool to assess the usability of assistive technology products and user satisfaction and make it accessible to everyone, including those with communication limitations/restritions, cognitive/mental deficits/etc. It would not be appropriate to talk about "neurological disorders" in general, as there are some persons with neurological diseases, but who do not present any difficulty in understanding and answering the existing questionnaires.
We appreciate the reviewer's comment and agree; however, this questionnaire was developed and tested by people with neurological diseases. It is right that some people do not present mental/cognitive disorders, and the questionnaire will be easier for them.

5. Why did you use the "Short Portable Mental Status Questionnaire for the Assessment of Organic Brain Deficit in Elderly Patients", since it is an instrument for the elderly population and the sample used ranged from 16 to 72 years (mean age 48 years)?

Thank you for your valuable observation. We agree with the reviewer's comment, and we chose the Pfeiffer test instead of MMSE because it is shorter, and we thought that should be too many questionnaires at the same time (Pfeiffer, Quest 2.0, and the new questionnaire).

6.Don't you think it is too short??!! Aren't the users still learning how to dela with the assistive device?
We thank the reviewer for this suggestion. We chose at least one month because we performed the study in an acute and subacute hospital. People usually stay in the hospital for between 3-6 months, depending on the severity of the pathology and if they are in a private or public insurance. When the people are autonomous, they are discharged. We choose only one month to perform the pre and the post before being discharged.
7. Explanation?
We appreciate the reviewer's comment. These patients did not use the AT at the moment of the interview; however, they used it in the past (last weeks, months)
We have added a sentence to clarify it. Please see table 1, pages 10 and 11, and below:

cNothing means that he/she did not use assistive technologies at the moment of the interview; however, they used them in the past.
8. Why from the acute?!
We appreciate the editor's suggestion. The hospitals where we performed the study were the Institut Guttmann (Badalona) and the Hospital de l'Esperança (Barcelona) in Spain; both specialized in acute and subacute patients.

9. Do you search for references/support? Please use a Reference.
Following the reviewer's suggestion, we have added a new reference. Please see page 14, line 270.
The statements of the questions are in first person to facilitate users' answers and usability experiences of the product subjectively [44].
First-person surveys in User Research | by Nikki Anderson | UX Collective. [cited 27 Oct 2022]. Available: https://uxdesign.cc/first-person-surveys-in-user-research-8732c5d9ab96

10. According to what?
We thank the reviewer's comment. We have clarified this information by adding a new sentence according to the comment. Please see page 14, line 272, and below

Powered by Editorial Manager® and ProduXion Manager® from Aries Systems Corporation

	Once the questionnaire was finished, ten users (see demographic characteristics in table 5) read and answered the questionnaire to know if all the requirements from the Delphi study were met.<br><br>11. It was a criterium for inclusion... Not here...<br>Following the reviewer's advice, we have removed this part from the text.<br><br>12. Initially the authors raised my expectation that they would develop a simple questionnaire to be used by people with neurological diseases and that there were no such resources adapted to you. How does this questionnaire differ from others?<br>We agree with the reviewer's comment. This questionnaire has been created from the responses of both health professionals specialized in neurology and patients with neurological disorders, so it has been designed based on a user-centered design, unlike the questionnaires that currently exist for the same purpose. In addition, the existing questionnaires, as I have already mentioned in the article, are either very long or include questions/items that are not relevant for all countries, as is the case with QUEST, which includes a part of after-sales service and technical issues that many people with diseases of neurological origin would not know how to respond. In addition, our questionnaire has included the relevant items for these people and why they decide to use or not use the AT product.<br><br>13.How does it differ from the health conditions that led to the other assistive technology tools?<br>We appreciate the reviewer's comment. In the sentence, we would like to state that in the study, we analyze different tools which have been analyzed by different people with different neurological conditions. Moreover, for these reasons, the sample has good heterogeneity.<br>REVIEWER 2<br>1.Can the authors elaborate a bit more the relationship between AT selection and outcome assessment?<br>Following your advice, we have added some sentences addressing your question. Please see page 4, line 81, and below:<br>For example, professionals prescribe assistive products without considering the user's needs. However, this clinical outcome assessment is important in clinical practice and research because it improves evidence and provides considerable feedback to healthcare professionals and patients, enhancing their empowerment with their opinions and needs and improving their quality of life[16].<br>2. Do you mean "usability of AT?". I would suggest you to add the word "usability" in this sentence.<br>When I read any paper on usability, I always would like to see a definition of usability. if you have space, please add a definition of usability so the readers would know "what usability" are you focusing on since you mentioned the QUEST's technical items are difficult so your questionnaire does not focus on technical part.<br>We appreciate the reviewer's comment. We have added the Usability term and its definition. Please see page 3, line 68 and below:<br>According to the ISO 9241-11 [9], usability is the extent to which specified users can use a product to achieve specified goals with effectiveness, efficiency, and satisfaction in a specified context of use. Understanding this, the design of a product…<br>3. I got a feeling some words are missing here. Please rephrase the sentence<br>We appreciate the reviewer's comment. We have rewritten the sentence. Please see page 4, line 91, and below:<br>There are other questionnaires developed exclusively to assess wheelchairs[22], and only a few are explicitly developed for assistive technologies. For example, the Quebec User Evaluation of Satisfaction with Assistive Technology (QUEST 2.0) [23] contains technical items such as weight, product dimensions, service delivery, repairs, and device services…<br>4. Can the authors clarify what you meant by limitations in the accessibility?<br>Following the reviewer's advice, we have modified the sentence. Please see page 5, lines 95 and below:<br>People with neurological diseases have limitations in the accessibility of the existing usability questionnaires, for example, in the comprehension of the questions or the answer form.

Powered by Editorial Manager® and ProduXion Manager® from Aries Systems Corporation

5. The recruitment of end users is an important part in this study. Now it is missing in the method. Please add in the recruitment process of end users.

We appreciate the reviewer's comment and have added a new sentence with this information. Please see page 5, line 114, and below:

Also, all the patients from the Institut Guttmann who voluntarily agreed to participate were interviewed to answer the questionnaire.
6. CAn you the authors elaborate what these values are or what they represent.
Following the reviewer's suggestion, we have added a new sentence. Please see page 7, line 141, and below:

Subsequently, the users (people with neurological diseases) read the questionnaire to assess their understanding of the questions and if their answers could be represented with the proposed response values (numbers, traffic light colors, and faces).
7. I do not understand this sentence and how this sentence is related to the sample. Do the participants have to pass the SPMSQ test in order to be eligible to fill in the questionnaire? Please rephrase the sentence.
Following the reviewer's suggestion, we have added a new sentence. Please see page 7, line 148, and below:
Exclusion criteria moderate to severe cognitive impairment based on the Pfeiffer SPMSQ test [32] translated into Spanish[33].
8. since you only use ref 33, it is more appropriate to mention the authors' last names and not just several authors.
We thank the reviewer's observation; we have corrected it and added the missing reference. Please see page 8, line 165, and below
According to different authors,[35,36] an interval between two days and two weeks between the test-retest interviews is recommended.

9. Please add which ICC model you have used, such as ICC 2.1 , ICC 3.1 etc. Please add it here and also in the results
We thank the reviewer's remark. According to this remark, we have added a new sentence. Please see page 8, line 172, and below.

Intraclass correlation coefficient (ICC) model 1.1 with a 95% of confidence interval (CI) was measured.
10. Please elaborate to your readers what do you mean by behaviour of items in the context of validity.
Following the reviewer's suggestion, we have added a new sentence. Please see page 9, line 190, and below:
In order to further analyze the construct validity of the different items, the Rasch model [41] was carried out.
11. this part of the analysis is an analysis of concurrent validity. Construct validity is an umbrella term for different validities.
My suggestion is to change this term to "concurrent validity".

Thank you for your valuable observation. Following the reviewer's suggestion, we have modified the term. Please see page 8, line 181, and below:
Concurrent validity. To know the relationship between the new questionnaire and another related questionnaire, QUEST 2.0[23] was chosen
12 . which model are you using? rating scale model?
We thank the reviewer's observation. We are using the Rasch model because it is commonly used in the analysis of the construct validity of an instrument. Please see the reference:
Boone WJ. Rasch Analysis for Instrument Development: Why, When, and How? CBE Life Sci Educ. 2016;15. doi:10.1187/CBE.16-04-0148

13. Move this sentence to the previous parapraph
Following the reviewer's suggestion, we have moved the sentence to the previous paragraph. Please see page 9, line 196 and below:

The study was developed following the COSMIN guidelines[42].
Ethical Approval
14. even though it is only 2 persons, it is not valid to ask end users that do not have

	any AT to answer the questions in the Delphi study, such as "would you change anything about our existing products?".<br><br>We thank the reviewer's observation. These patients didn't use the AT at the moment of the interview; however, they have used it in the past (last weeks, months)<br>We will add a sentence to clarify it. Please see table 1 on page 11 and below:<br><br>cNothing means that he/she did not use assistive technologies at the moment of the interview; however, they used it in the past<br>15.Did these experts involve in the rounds? Please clarify.<br>Following the reviewer's advice, we have added a new sentence. Please see page 12, line 232, and below<br>Thirty-four experts from the Delphi study from the private neurorehabilitation hospital of acute neurological patients and the public hospital of acute stroke patients were selected to evaluate the items obtained from round two.<br>16. Ergonomics is related to body posture and positions, often in workplace. My daily live needs is much bigger than body posture. Please explain why this item is categorized as ergonomics?<br>We thank the reviewer for this suggestion. According to the ISO 9241-11:2018(en), Ergonomics of human-system interaction — Part 11: Usability: Definitions and concepts, ergonomics/human factor is a scientific discipline concerned with the understanding of interactions among humans and other elements of a system, and the profession that applies theory, principles, data, and methods to design in order to optimize human well-being and overall system performance,<br>Another definition: Ergonomics is the design and engineering of human-machine systems to enhance human performance.<br>Dempsey, Patrick G., Wogalter, Michael S., & Hancock, Peter A. (2000). What is in a name? Using terms from definitions to examine the fundamental foundation of human factors and ergonomics science. Theoretical Issues in Ergonomics Science, 1(1), 3-10.<br>Also, see the text below from Ahasan, R., Campbell, D., Salmoni, A., & Lewko, J. (2001). HFs/ergonomics of assistive technology. Journal of physiological anthropology and applied human science, 20(3), 187–197. https://doi.org/10.2114/jpa.20.187.<br>How ergonomic designs could be helpful for special needs, Coleman and Pullinger (1993) noted some aspects of improvement features of disability and the aging process. Ergonomics application is important in the design and development of AT towards meeting the needs of disabled people. Hence by addressing the actual needs of these people, ergonomics principles are to be followed to enhance the mobility and communication of disabled people. Ergonomically designed AT usually enhance functional requirements that correctly fit the future application of the users meeting the needs of disabled people.<br><br>For these reasons, we thought that ergonomics fit in this category.<br><br>17. Please provide information about these ten users? were they in the Delphi rounds? do you do AT? what are their diagnoses?<br>We appreciate the reviewer's comment. We have added a new sentence in order to clarify it. Please see page 14, line 271.<br>Once the questionnaire was finished, ten users (see demographic characteristics in table 5)<br>Also, we have added a new table with the demographic data of the ten users. Please see page 15, line 274, and below:<br>Table 5. Demographic characteristics users from the questionnaire design.<br>Questionnaire analysisn= 10<br>Demographic CharacteristicsNumberPercentage<br>Age<br>Between 18 and 25 yearsn=1 10%<br>Between 26 and 35 yearsn=220%<br>Between 36 and 45 yearsn=220%<br>Between 46 and 65 yearsn= 330%<br>More than 65 yearsn= 220%<br>Sex<br>Femalen=660%<br>Malen=440%<br>Pathology

Powered by Editorial Manager® and ProduXion Manager® from Aries Systems Corporation

	Spinal Cord Injuryn=440%<br>Traumatic Brain Injuryn= 220%<br>Stroken= 330%<br>Othern= 110%<br>Assistive Technologies<br>Wheelchairn= 880%<br>Canesn= 110%<br>Orthesisn=440%<br>Othern=110%<br>Nothingan=110%<br><br>aNothing means that he/she did not use assistive technologies at the moment of the interview; however, they used it in the past<br><br>18. What is a usability test? please clarify<br>We thank the reviewer's observation, and we have added a new sentence in the introduction to clarify it. Please see page 4, line 73 and below:<br>For that reason, it is essential to perform a usability test during product development to know if it fits the user's needs. Usability testing refers to evaluating a product or service by testing it with representative users [11].<br><br><br>19. did these 51 patients not the end users in the Delphi study? if not, please replace the word "round" with " adminstration" or " occasion".<br>We agree with the reviewer's comment. We have changed the word "round" to "administration" to distinguish. Also, we expressed that the patients are different from the Delphi study. Please see page 16, line 285, and below:<br>A total of 51 people with neurological diseases consecutive recruited from the Institut Guttmann Hospital voluntarily agreed to participate in the study. These people were different from the Delphi study, and their demographic characteristics are summarized in Table 6.<br><br>20. Brain injury is often acquired or traumatic. What are the brain injuries in these 4 patients?<br>We thank the reviewer's observation, and we have corrected it. Please see table 6 on page 16 and below:<br>Table 6. Demographic characteristics of the 51 participants in the reliability and validity of NATU Quest.<br>Demographic CharacteristicsMean /NumberPercentage<br>Age, range 16-72 yearsMean = 48<br><br>Sex<br>Femalen=2039%<br>Malen= 3161%<br><br>Pathology<br>Spinal cord Injuryn=2243%<br>Traumatic Brain Injuryn=36%<br>Stroken=1631%<br>Acquired Brain injuryn=48%<br>Other neurological diseasesn=612%<br><br>Education<br>Secondaryn=1325%<br>High School Diploman=12%<br>Certificate of Professional Standardsn=1529%<br>Higher Level Education Cycle n=714%<br>University Degreen=1530%<br><br>Questionnaire form<br>Self-administrationn=2453%<br>Interviewn=2747%<br><br>Assistive Technologies

Wheelchairn=29
Walking stickn=8
Walkern=3
Splintn=7
Treadmilln=5

21.Cognitive impairments are common in persons with neurological diseases. Do you have a criterion that the end users do not have cognitive impairments?
And Pfeiffer test was not mentioned at all in the method. Please add this test in the method section.
We appreciate the reviewer's comment. Pfeiffer test was mentioned in the inclusion/exclusion criteria. Please see page 7, line 148, and below:
Exclusion criteria moderate to severe cognitive impairment based on the Pfeiffer SPMSQ test [32] translated into Spanish[33].
22.a question about the expertise of doctors and PTs in assistive technology. Do doctors work with AT ? it is often occupational therapists and physiotherapists who have knowledge. Please add more information about doctors' expertise in AT in your country.
We thank the reviewer's comment. We have exchanged medical doctors with occupational therapists to make it more understandable. However, in the center where we performed the study, there is a transdisciplinary team, and the prescription of the orthoses is agreed upon by medical doctors, PT, occupational therapists, and orthopaedists. Please see page 18, line 325 and below:
The items included in the questionnaire, the format of the questionnaire, and the answer form were derived through two rounds of a Delphi study[27] based on the opinion of 69 experts (neurorehabilitation professionals, such as occupational therapists and physiotherapists) and 63 users (people with neurological diseases).
23. do you know if the other regions in Spain have similar experiences?
Following the reviewer's suggestion, we have added a new sentence. Please see page 19, line 343, and below:
(1) Selection bias of the participants since most of them were from the same province. However, other regions have the same experiences. [45]
24. no information on Pfeiffer test in the study.
We appreciate the reviewer's comment. Pfeiffer test was mentioned in the inclusion/exclusion criteria. Please see page 7, line 148, and below:
Exclusion criteria moderate to severe cognitive impairment based on the Pfeiffer SPMSQ test [32] translated into Spanish[33].

25. the ATs in your study are mobility AT. Do you want to measure only mobility AT or other AT that support cognition? if yes, please add more information.
We thank the reviewer's observation. We only wanted to measure mobility AT. Others, such as cognition or audition, have other parameters and items.

26. I don't see if your test-retest reliability has compared with Quest 2. Please clarify.
We thank the reviewer's observation. Please see the results from pages 15 to 17 and in table 7 and below:
Reliability and Validity
Sample description
A total of 51 people with neurological diseases consecutive recruited from the Institut Guttmann Hospital voluntarily agreed to participate in the study. These people were different from the Delphi study, and their demographic characteristics are summarized in Table 6. Fifty-three percent of the participants answered the questionnaire through an interview due to physical limitations, and the rest (47%) answered it by themselves. First, the participants answered the new questionnaire and the QUEST 2.0. On average, the participants completed the new questionnaire within 102.40 seconds in the first administration and within 82.08 seconds in the second administration. The QUEST 2.0 was administered once before the NATU Quest, and the participants needed an average of 74 seconds to complete it. Participants scored an assistive product they had used in the last three months. All the patients answered all the items.
Table 6. Demographic characteristics of the 51 participants in the reliability and validity of NATU Quest.
Demographic CharacteristicsMean /NumberPercentage
Age, range 16-72 yearsMean = 48

Powered by Editorial Manager® and ProduXion Manager® from Aries Systems Corporation

	Sex<br>Femalen=2039%<br>Malen= 3161%<br><br>Pathology<br>Spinal cord Injuryn=2243%<br>Traumatic Brain Injuryn=36%<br>Stroken=1631%<br>Acquired Brain injuryn=48%<br>Other neurological diseasesn=612%<br><br>Education<br>Secondaryn=1325%<br>High School Diploman=12%<br>Certificate of Professional Standardsn=1529%<br>Higher Level Education Cycle n=714%<br>University Degreen=1530%<br><br>Questionnaire form<br>Self-administrationn=2453%<br>Interviewn=2747%<br><br>Assistive Technologies<br>Wheelchairn=29<br>Walking stickn=8<br>Walkern=3<br>Splintn=7<br>Treadmilln=5<br><br>Reliability Results<br>The internal consistency reliability of the NATU Quest was analyzed using the Cronbach's Alpha[34] (α=0.895). This result can be interpreted as good reliability. Reliability through test-retest. A retest was performed 15 days after answering the two questionnaires for the first time to assess the reliability of the NATU Quest. Table 7 shows the weighted quadratic Kappa coefficient and Spearman's coefficient results of the NATU Quest. The results showed a moderate to considerable concordance between NATU Quest items test-retest in the Kappa coefficient because all the results were above 0,50. The results also showed a strong association between test-retest in the Spearman's coefficient (ρ = 0.818), significant with p-value < 0.0001. The results of the ICC showed good reliability (ICC = 0.869; CI 95% 0.781 to 0.923).<br>Table 7. NATU Quest reliability of the test-retest.<br>NATU Quest<br>ItemsWeighted Kappa95% Confidence Interval<br>Lower LimitUpper Limit<br>1Effectiveness0.7270.5350.918<br>2Comfortable0.7330.6450.821<br>3Adaptability0.5850.4340.735<br>4Easy to put on/off0.6310.3990.863<br>5Safe0.5510.2060.895<br>6Functionality0.6850.5410.828<br>7Ergonomics0.6730.5070.840<br>8Easy to use0.5440.3320.755<br>9Easy to remember how to use it0.6240.2980.950<br>10Satisfaction0.7140.5570.871<br><br>Spearman 0.818a<br>aThe correlation is significant p< 0.0001 (bilateral).<br>Concurrent validity<br>The correlation of the total scores between NATU Quest and QUEST 2.0 analyzed with the Spearman's coefficient was strong with ρ = 0.756 significant, with p-value < 0.0001
**Additional Information:**	
**Question**	**Response**

Powered by Editorial Manager® and ProduXion Manager® from Aries Systems Corporation

**Financial Disclosure**<br><br>Enter a financial disclosure statement that describes the sources of funding for the work included in this submission. Review the submission guidelines for detailed requirements. View published research articles from *PLOS ONE* for specific examples.<br><br>This statement is required for submission and **will appear in the published article** if the submission is accepted. Please make sure it is accurate.<br><br>**Unfunded studies**<br>Enter: *The author(s) received no specific funding for this work.*<br><br>**Funded studies**<br>Enter a statement with the following details:<br>• Initials of the authors who received each award<br>• Grant numbers awarded to each author<br>• The full name of each funder<br>• URL of each funder website<br>• Did the sponsors or funders play any role in the study design, data collection and analysis, decision to publish, or preparation of the manuscript?<br>• **NO** - Include this sentence at the end of your statement: *The funders had no role in study design, data collection and analysis, decision to publish, or preparation of the manuscript.*<br>• **YES** - Specify the role(s) played.<br><br>* typeset	The funders had no role in study design, data collection and analysis, decision to publish, or preparation of the manuscript.
**Competing Interests**<br><br>Use the instructions below to enter a competing interest statement for this submission. On behalf of all authors, disclose any competing interests that could be perceived to bias this work—acknowledging all financial support and any other relevant financial or non-financial competing interests.<br><br>This statement is required for submission	The authors have declared that no competing interests exist.

and **will appear in the published article** if the submission is accepted. Please make sure it is accurate and that any funding sources listed in your Funding Information later in the submission form are also declared in your Financial Disclosure statement.<br><br>View published research articles from *PLOS ONE* for specific examples.<br><br>**NO authors have competing interests**<br><br>Enter: *The authors have declared that no competing interests exist.*<br><br>**Authors with competing interests**<br><br>Enter competing interest details beginning with this statement:<br><br>*I have read the journal's policy and the authors of this manuscript have the following competing interests: [insert competing interests here]*<br><br>* typeset	
**Ethics Statement**<br><br>Enter an ethics statement for this submission. This statement is required if the study involved:<br><br>• Human participants<br>• Human specimens or tissue<br>• Vertebrate animals or cephalopods<br>• Vertebrate embryos or tissues<br>• Field research<br><br>Write "N/A" if the submission does not require an ethics statement.<br><br>General guidance is provided below. Consult the submission guidelines for detailed instructions. **Make sure that all information entered here is included in the Methods section of the manuscript.**	N/A

**Format for specific study types**<br><br>**Human Subject Research (involving human participants and/or tissue)**<br>• Give the name of the institutional review board or ethics committee that approved the study<br>• Include the approval number and/or a statement indicating approval of this research<br>• Indicate the form of consent obtained (written/oral) or the reason that consent was not obtained (e.g. the data were analyzed anonymously)<br><br>**Animal Research (involving vertebrate animals, embryos or tissues)**<br>• Provide the name of the Institutional Animal Care and Use Committee (IACUC) or other relevant ethics board that reviewed the study protocol, and indicate whether they approved this research or granted a formal waiver of ethical approval<br>• Include an approval number if one was obtained<br>• If the study involved *non-human primates*, add *additional details* about animal welfare and steps taken to ameliorate suffering<br>• If anesthesia, euthanasia, or any kind of animal sacrifice is part of the study, include briefly which substances and/or methods were applied<br><br>**Field Research**<br><br>Include the following details if this study involves the collection of plant, animal, or other materials from a natural setting:<br>• Field permit number<br>• Name of the institution or relevant body that granted permission	
**Data Availability**<br><br>Authors are required to make all data underlying the findings described fully available, without restriction, and from the time of publication. PLOS allows rare exceptions to address legal and ethical concerns. See the PLOS Data Policy and FAQ for detailed information.	Yes - all data are fully available without restriction

A Data Availability Statement describing where the data can be found is required at submission. Your answers to this question constitute the Data Availability Statement and **will be published in the article**, if accepted.<br><br>**Important:** Stating 'data available on request from the author' is not sufficient. If your data are only available upon request, select 'No' for the first question and explain your exceptional situation in the text box.<br><br>Do the authors confirm that all data underlying the findings described in their manuscript are fully available without restriction?	
**Describe where the data may be found in full sentences. If you are copying our sample text, replace any instances of XXX with the appropriate details.**<br><br>• If the data are **held or will be held in a public repository**, include URLs, accession numbers or DOIs. If this information will only be available after acceptance, indicate this by ticking the box below. For example: *All XXX files are available from the XXX database (accession number(s) XXX, XXX.*).<br>• If the data are all contained **within the manuscript and/or Supporting Information files**, enter the following: *All relevant data are within the manuscript and its Supporting Information files.*<br>• If neither of these applies but you are able to provide **details of access elsewhere**, with or without limitations, please do so. For example:<br><br>*Data cannot be shared publicly because of [XXX]. Data are available from the XXX Institutional Data Access / Ethics Committee (contact via XXX) for researchers who meet the criteria for access to confidential data.*<br><br>*The data underlying the results presented in the study are available from (include the name of the third party*	All relevant data are within the manuscript and its Supporting Information files.

and contact information or URL).<br>• This text is appropriate if the data are owned by a third party and authors do not have permission to share the data.<br><br>* typeset	
Additional data availability information:	Tick here if the URLs/accession numbers/DOIs will be available only after acceptance of the manuscript for publication so that we can ensure their inclusion before publication.

Powered by Editorial Manager® and ProduXion Manager® from Aries Systems Corporation

Manuscript Click here to access/download;Manuscript;Manuscript.doc

Developing An Assistive Technology Usability Questionnaire for People with Neurological Diseases

Maria Masbernat-Almenara[1,2,3]; Francesc Rubi-Carnacea [1,2,3*]; Eloy Opisso [4,5,6]; Esther Duarte-Oller [7, 8]; Josep Medina-Casanovas [4,5,6;] Fran Valenzuela-Pascual[1,2,3]

[1] Department of Nursing and Physiotherapy, University of Lleida, Lleida, Spain

[2] Research group of health care. IRB Lleida, Institute for Biomedical Research Dr. Pifarré Foundation, Lleida, Spain

[3] Group on Society Studies, Health, Education and Culture, University of Lleida, Spain

[4] Institut Guttmann, Neurorehabilitation Institute, Badalona, Spain

[5] Universitat Autònoma de Barcelona, Cerdanyola del Vallés, Barcelona, Spain

[6] Fundació Institut d'investigació en Ciències de la Salut Germans Trias i Pujol, Badalona, Barcelona, Spain

[7] Physical Medicine and Rehabilitation Department, Parc de Salut Mar (Hospital del Mar, Hospital de l'Esperança). Barcelona, Catalonia, Spain

[8] Rehabilitation Research Group, Hospital del Mar Medical Research Institute (IMIM). Barcelona, Catalonia, Spain.

*Corresponding Author:

Francesc Rubí Carnacea, Ph.D,

Email: francesc.rubi@udl.cat

Abstract

Purpose. This study describes the development of a questionnaire for assessing the usability of assistive technologies accessible to people with neurological diseases. *Methods.* A Delphi study was conducted to identify relevant items for the questionnaire. After that, the content validity was addressed to identify the essential items. Once the questionnaire was designed following the results of the Delphi study and content validity, the reliability, validity, and the Rasch model of the questionnaire were examined. *Results*. Two rounds of the Delphi study were carried out. A total of 73 participants (42 experts and 31 users) participated in round 1, and 59 people (27 experts and 32 users) in round 2. A total of 53 and 29 items were identified in rounds 1 and 2, respectively. In the content validity, we found nine items above the threshold of 0.58. Finally, ten items were included in the questionnaire. Fifty-one participants participate in the reliability and validity of the questionnaire. The internal consistency reliability of the questionnaire analyzed by Cronbach's Alpha was $\alpha=0,895$. There was moderate to considerable concordance among our questionnaire items test-retest in the Kappa coefficient and a strong association between test-retest in the Spearman's coefficient $\rho=0.818$ ($p<0,001$). The intraclass correlation coefficient was 0,869 with a 95% confidence interval (0,781;0,923). There was a strong correlation between the total scores of the new questionnaire and other validated questionnaires analyzed with Spearman's coefficient $\rho=0.756$ ($p<0,001$). The ten items demonstrated a satisfactory fit to the Rasch model. *Conclusions*. The present study suggested that the new questionnaire is a reliable 10-item usability questionnaire that allows subjective and quick assessment of the usability of assistive technologies by people with neurological diseases.

Key words: Nervous System Diseases, Neurological Rehabilitation, Questionnaire Design, Self-Help Devices, User-centered Design

Introduction

Neurological disorders are among the most important causes of disability (247–308 million) and death (8·8–9·4 million) worldwide. [1]In addition, the burden of neurological disorders in public health has increased substantially in the last 25 years because of expanding population numbers, aging, and increased survivor rates from stroke and other neurological disorders.[2] These survivors require intensive rehabilitation to reduce the sequelae of the disorders, increase their quality of life, and improve their autonomy in activities of daily living. In most cases, some assistive technology is needed.

Recent advances in technology and rehabilitation have led to the development of new tools to assess people with disabilities and improve their functionality and autonomy in their daily life.[3] It is known that a good acceptance of assistive technology can improve the quality of life and social inclusion of these patients[4]. However, not all products achieve the goal for which they were designed since they often do not consider the real needs of users.[5] Therefore, it is increasingly necessary to involve end-users from the beginning of developing new products.[6] A product can only be considered successful if it is used, and currently, more than 50% of users abandon their new products because they are not sufficiently usable.[7,8] For this reason, usability is becoming more critical in engineering and rehabilitation. According to the ISO 9241-11 [9], usability is the extent to which specified users can use a product to achieve specified goals with effectiveness, efficiency, and satisfaction in a specified context of use. Understanding this, the design of a product should not involve only multidisciplinary theoretical foundations, but user

experience should be considered throughout the design process to ensure quality, improve usability, and increase product acceptability.[5,10] For that reason, it is essential to perform a usability test during product development to know if it fits the user's needs. Usability testing refers to evaluating a product or service by testing it with representative users [11].

According to the WHO, assistive technology is a general term covering the systems and services related to delivering assistive products and services.[12] Assistive products aim to maintain or improve an individual's functioning and independence, promoting their well-being.[12]

There is a lack of evidence-based procedures for assistive technologies selection [13–15]. For example, professionals prescribe assistive products without considering the user's needs. However, this clinical outcome assessment is important in clinical practice and research because it improves evidence and provides considerable feedback to healthcare professionals and patients, enhancing their empowerment with their opinions and needs and improving their quality of life[16].

Many existing usability questionnaires have been developed to evaluate software usability and web accessibility[17] and not assistive technologies [18]. Some questionnaires[19–21] measure the psychosocial impact of quality of life by using assistive technologies from the point of view of people with disabilities. Those are interesting questionnaires; however, they do not evaluate the usability of assistive technologies per se. There are other questionnaires developed exclusively to assess wheelchairs[22], and only a few are explicitly developed for assistive technologies. For example, the Quebec User Evaluation of Satisfaction with Assistive Technology (QUEST 2.0) [23] contains technical items such as weight, product dimensions, service delivery,

repairs, and device services. People with neurological diseases have limitations in the accessibility of the existing usability questionnaires, for example, in the comprehension of the questions or the answer form. In addition, existing questionnaires are too extensive for them or do not address all the essential items for assessing usability and user satisfaction.[18,24] It is known that longer questionnaires incur high costs in data collection and reduce the number of answers due to the time answering and the quality of the information gathered due to fatigue.[25,26]

The objective of this study was to develop a short questionnaire focused only on the usability of assistive technology products and user satisfaction, and it should be accessible and easy to understand by people with neurological diseases.

Materials and methods

Questionnaire Design

Delphi study

To construct an easy-to-use questionnaire, we performed a Delphi study [27], as it is a prospective approach that seeks to derive a consensus from a group of experts based on the analysis and reflection of a defined problem. A google form questionnaire was sent via email to the neurological healthcare professionals from the private neurorehabilitation hospital of acute neurological patients (Institut Guttmann, Spain) and the public hospital of acute stroke patients (Hospital de l'Esperança, Spain). Also, all the patients from the Institut Guttmann who voluntarily agreed to participate were interviewed to answer the questionnaire.

The questionnaire contains six questions: three were open questions related to assistive technologies, and three were multiple-choice questions about how a questionnaire should be easy and quick to answer. The three open questions were: (1) List some requirements or characteristics important to how the assistive products should or should be manufactured. (2) Would you change anything about the existing assistive products? (3) What kind of assistive products would you like to have? (for users)/ in your workplace? (experts). The three multiple-choice questions related to the questionnaires were: (1) the time required for you to answer a questionnaire, (2) which questionnaire format the user preferred, and (3) how many questions you would like to answer.

Content Validity Ratio

Once the Delphi study finished, and the items were obtained, they were evaluated using the content validity ratio (CVR).[28,29] Content validity addresses the degree to which items of an instrument sufficiently represent the content domain and answers the question of to what extent the selected sample in an instrument or instrument items is a comprehensive sample of the content. [29] The content validity ratio varies between 1 and -1. Higher scores indicate greater agreement by panel members on the need for an item in an instrument. The formula of content validity ratio is CVR= (Ne - N/2)/(N/2), in which the Ne is the number of panelists indicating "essential" and N is the total number of panelists.[29] The numeric value of the content validity ratio is determined by Lawshe Table.[28]

Questionnaire design

The questions were written based on the qualitative data obtained in the Delphi study and the items obtained in the CVR. Subsequently, the users (people with neurological

diseases) read the questionnaire to assess their understanding of the questions and if their answers could be represented with the proposed response values (numbers, traffic light colors, and faces).

Questionnaire reliability and validity

The reliability and validity of the new questionnaire were addressed.

Sample size. The recommended sample size for similar studies has been established as 50 subjects.[30,31] The inclusion criteria for participants were (1) adults (≥18 years old) with neurological diseases that have been using some assistive products for the past month. Exclusion criteria moderate to severe cognitive impairment based on the Pfeiffer SPMSQ test [32] translated into Spanish[33].

Assistive product analyzed: It is challenging to analyze the same product because neurological patients have personalized assistive technologies adapted to their disability. Therefore, all the participants could choose which product addressed, only with the condition that they had used it for at least one month.

Data analysis of Reliability

The reliability and validity of the new questionnaire and all the data were analyzed using the SPSS Statistics 27 program.

The internal consistency reliability of the new questionnaire was analyzed using Cronbach's Alpha.[34] The value of the coefficients was interpreted as follows: unacceptable ($0.5 \geq \alpha$), poor ($0.6 \geq \alpha \geq 0.5$), questionable ($0.7 \geq \alpha \geq 0.6$), acceptable ($0.8 \geq \alpha \geq 0.7$). good ($0.9 \geq \alpha \geq 0.8$), and excellent $\alpha \geq 0.9$).[34]

Test-retest. Reliability can be assessed with a test-retest comparison. This method evaluates the stability of the results at two different points in time using a stability coefficient. To perform the test-retest, the same questionnaire was administered on two occasions, separated by a certain period, to the same subjects and under the same conditions. According to different authors,[35,36] an interval between two days and two weeks between the test-retest interviews is recommended. Accordingly, the interval between the two tests was set to 15 days. In order to know the concordance between the test and the retest, data was analyzed by weighted quadratic Kappa coefficient. [37] The value of the coefficients was interpreted as follows: poor (< 0.00), weak (0.00–0.20), good (0.20–0.40), moderate (0.41–0.60), considerable (0.61–0.80), and almost perfect (0.81–1.00).[37]

Intraclass correlation coefficient (ICC) model 1.1 with a 95% of confidence interval (CI) was measured. ICC indicates the degree to which the participants maintained their opinion during the test-retest.[38] The value of the coefficients was interpreted as follows: poor reliability (< 0.5), moderate reliability (0.5–0.75), good reliability (0.75–0.9), and excellent reliability (> 0.90).[38]

The normality of the data was tested using the Kolmogorov-Smirnov test, and the repeatability was determined by the Spearman's correlation coefficient[39] between the test and the retest.

Data Analysis of validity

Concurrent validity. To know the relationship between the new questionnaire and another related questionnaire, QUEST 2.0[23] was chosen. It is the shortest worldwide questionnaire and has been translated and validated in several languages. Only eight items

of the QUEST 2.0 was administered to users in Spanish[40] because the other four items are related to services. Also, in this study, data was tested using the Kolmogorov-Smirnov test and analyzed using Spearman's correlation coefficient [39].

Measurement of other parameters. The time was measured while the participants answered the questionnaire to obtain the average time of all the participants.

Rasch Model

In order to further analyze the construct validity of the different items, the Rasch model [41] was carried out. This model gives an idea of the scale's internal consistency by relating the item's difficulty to the person's ability[41]. The values of infit and outfit mean squares (MNSQ) of 1 indicate a perfect fit between the data and the model, values between 0.5 and 1.5 indicate an acceptable fit, and values greater than 2 indicate a severe mismatch[41].

The study was developed following the COSMIN guidelines[42].

Ethical Approval

The Institut Guttmann Neurorehabilitation Hospital Ethics Committee approved this study. In addition, this research was conducted following the Declaration of Helsinki's ethical principles. All participants participated voluntarily, and they signed an informed written consent form. Their personal data were archived following the Spanish Organic Law 3/2018, December 5, on the Protection of Personal Data and guarantee of digital rights.

Results

Study Delphi

Two rounds of a Delphi study were needed to obtain all the items of the new questionnaire. A total of 73 participants (42 experts and 31 users) were involved in round 1 (Table 1). From round 1, 53 different items and qualitative information about questionnaires were derived qualitatively.

Round two involved 59 people (27 experts and 32 users) (Table 1). This round obtained 15 items and data about the scale. The items were: "effectiveness", "comfort", "adaptability", "easy to put on/off", "safe", "lightweight", "functionality", "ergonomic", "economical", "affordable", "easy to use", "feedback", "stimulating," "monitored" and "movement facilitator. Due to "economical" and "affordable" have the same meaning, both were considered as a single item. Additionally, in this round and following the usability premises, the items "aesthetics" and "easy to remember how to use it" were added. Finally, 16 items were analyzed using the content validity ratio.

Table 1. Demographic Characteristics of participants of the two rounds in the Delphi Study.

**EXPERT PANEL**	**Round 1 n=42**		**Round 2 n= 27[a]**	
**Demographic Characteristics**	**Number**	**Percentage**	**Number**	**Percentage**
**Age**				
Between 18 and 25 years	n= 3	7%	n=1	4%
Between 26 and 35 years	n=17	41%	n=11	41%
Between 36 and 45 years	n=11	26%	n=8	29%
Between 46 and 65 years	n= 11	26%	n=7	26%
More than 65 years	n= 0	0%	n=0	0%
**Sex**				
Female	N=30	71%	19	70%

Male	N=12	29%	8	30%
**Profession**				
Physician	n=9	21%	6	22%
Physiotherapist	n=14	33%	9	33%
Occupational Therapist	n=13	31%	8	30%
Other[b]	n=6	15%	4	15%
**Expert in Neurorehabilitation**				
Yes	39	93%	27	100%
**USER PANEL**	**Round 1 n= 31**		**Round 2 n= 32[d]**	
**Demographic Characteristics**	**Number**	**Percentage**	**Number**	**Percentage**
**Age**				
Between 18 and 25 years	n= 4	13%	n=1	3%
Between 26 and 35 years	n=6	19%	n=9	28%
Between 36 and 45 years	n=5	16%	n=3	10%
Between 46 and 65 years	n= 13	42%	n=16	50%
More than 65 years	n= 3	10%	n=3	9%
**Sex**				
Female	n=17	45%	n=16	50%
Male	n=14	55%	n=16	50%
**Pathology**				
Spinal Cord Injury	n=13	42%	n=7	22%
Traumatic Brain Injury	n=5	16%	n=1	3%
Stroke	n=8	26%	n=13	41%
Other	n=5	16%	n=11	34%
**Assistive Technologies**				
Wheelchair	n= 23	53%	n=19	36%
Canes	n= 8	18%	n=12	23%
Orthesis	n=8	19%	n=15	28%
Other	n=2	5%	n=6	11%
Nothing[c]	n=2	5%	n=1	2%

[a]In the second round, 36% did not answer the questionnaire. [b]Others are, for example, orthopedic professionals and engineers. [c]Nothing means that he/she did not use assistive technologies at the moment of the interview; however, they used them in the past. [d]Users are different from round 1.

Content Validity Ratio (CVR)

Thirty-four experts from the Delphi study from the private neurorehabilitation hospital of acute neurological patients and the public hospital of acute stroke patients were selected to evaluate the items obtained from round two. 70% of the participants have more than ten years of expertise in neurorehabilitation, and all of them used to work with assistive technologies experts (Table 2).

Table 2. Demographic Characteristics of participants in Content Validity.

	**Content Validity n=34**	
**Demographic Characteristics**	**Number**	**Percentage**
**Age**		
Between 26 and 35 years	n=8	24%
Between 36 and 45 years	n=12	35%
Between 46 and 65 years	n= 14	41%
**Sex**		
Female	N=21	62%
Male	N=13	38%
**Profession**		
Physician	n= 11	32%
Physiotherapist	n=14	41%
Occupational Therapist	n=7	21%
Other[a]	n=2	6%
**Expert in Neurorehabilitation[b]**		
Between 1 and 5 years	n=5	15%
Between 5 and 10 years	n=5	15%
Between 10 and 20 years	n=12	35%
More than 20 years	n=12	35%

[a]Others are, for example, orthopedic professionals and engineers. [b]All the participants used to work with people with neurological diseases.

Participants had to choose which items were *essential*, *useful but not essential*, and *not essential* when evaluating the items. Table 3 shows the results. Nine of the sixteen items exceeded the threshold of 0.58. In addition, the experts agreed to accept the item "comfortable" because its threshold was 0.53.

Table 3. Content Validity Ratio results.

**Items**	**ne**	**N**	**CVR[a]**
1. Effectiveness	33	34	0,97
2. Comfortable	18	34	0,53
3. Adaptability	21	34	0,62
4. Easy to put on/off	20	34	0,59
5. Safe	32	34	0,94
6. Lightweight	12	34	0,35
7. Functionality	31	34	0,91
8. Ergonomic	22	34	0,65
9. Economical/ Affordable	13	34	0,38
10. Easy to use	24	34	0,71
11. Aesthetics	4	34	0,12
12. Easy to remember how to use it	20	34	0,59
13. Feedback	16	34	0,47
14. Stimulating	14	34	0,41
15. Monitored	10	34	0,29
16. Movement Facilitator	21	34	0,62

[a]CVR (content validity ratio) = (Ne-N/2) / (N/2) with 34 people in the expert panel (N=34), the items with a CVR bigger than 0.58 (in bold) will remain at the instrument, and the rest will be eliminated.

The experts considered "functional" and "movement facilitator" as one word due to the similarity of their meanings. The item "satisfaction" was added to the questionnaire to ascertain the end-user opinion of the product. Therefore, ten items were selected to create the questionnaire (Table 4).

Table 4. Questions and the items that are involved in each question.

**Questions**	**Items**

1	I believe that _____ can help me improve my functional independence	Effectiveness
2	I feel comfortable wearing/using ____	Comfortable
3	_____ adapts to my characteristics and needs	Adaptability
4	Donning/Doffing …………….. is quick and easy for me.	Easy to put on / off
5	I feel safe using/wearing _____ / _____ is safe in its use	Safe
6	_____ allows me to achieve my goal/ allows me to perform a movement/action that I could not do before	Functionality/ movement facilitator
7	_____ adapts to my daily live needs	Ergonomics
8	In general, _____ is easy to use	Easy to use
9	Information and instructions of use______ are easy to understand and easy to remember	Easy to remember how to use it
10	Overall, I am satisfied with______	Satisfaction

Questionnaire design

Following the qualitative information from the Delphi study, the questionnaire was formulated in an understandable language, and the length was as short as possible. When necessary, the users could fill the blank space with the product's name being evaluated. The 6-point Likert scale was chosen since it forces the respondent to decide positively or negatively according to the item in question[43]. A numeric panel from 0 to 5 points was used, and each number was associated with a box using the colors of the traffic light to facilitate the choice. Furthermore, faces with expressions were added to facilitate the answers according to the patients with neurological diseases responses from the Delphi study.

The statements of the questions are in first person to facilitate users' answers and usability experiences of the product subjectively [44].

Once the questionnaire was finished, ten users (see demographic characteristics in table 5) read and answered the questionnaire to know if all the requirements from the Delphi study were met. During this process, some wording modifications were made.

Table 5. Demographic characteristics users from the questionnaire design.

**Questionnaire analysis**	**n= 10**	
**Demographic Characteristics**	**Number**	**Percentage**
**Age**		
Between 18 and 25 years	n=1	10%
Between 26 and 35 years	n=2	20%
Between 36 and 45 years	n=2	20%
Between 46 and 65 years	n= 3	30%
More than 65 years	n= 2	20%
**Sex**		
Female	n=6	60%
Male	n=4	40%
**Pathology**		
Spinal Cord Injury	n=4	40%
Traumatic Brain Injury	n= 2	20%
Stroke	n= 3	30%
Other	n= 1	10%
**Assistive Technologies**		
Wheelchair	n= 8	80%
Canes	n= 1	10%
Orthesis	n=4	40%
Other	n=1	10%
Nothing[a]	n=1	10%

[a]Nothing means that he/she did not use assistive technologies at the moment of the interview; however, they used it in the past

The new questionnaire was named "Assistive Technology Usability Questionnaire for people with Neurological diseases" (NATU Quest) and included ten questions. It should be administered at the end of a usability test. The final version of the questionnaire, questionnaire score, and interpretation are available in the supporting information file.

Reliability and Validity

Sample description

A total of 51 people with neurological diseases consecutive recruited from the Institut Guttmann Hospital voluntarily agreed to participate in the study. These people were different from the Delphi study, and their demographic characteristics are summarized in Table 6. Fifty-three percent of the participants answered the questionnaire through an interview due to physical limitations, and the rest (47%) answered it by themselves. First, the participants answered the new questionnaire and the QUEST 2.0. On average, the participants completed the new questionnaire within 102.40 seconds in the first administration and within 82.08 seconds in the second administration. The QUEST 2.0 was administered once before the NATU Quest, and the participants needed an average of 74 seconds to complete it. Participants scored an assistive product they had used in the last three months. All the patients answered all the items.

Table 6. Demographic characteristics of the 51 participants in the reliability and validity of NATU Quest.

**Demographic Characteristics**	**Mean /Number**	**Percentage**
Age, range 16-72 years	Mean = 48	
**Sex**		
Female	n=20	39%
Male	n= 31	61%
**Pathology**		
Spinal cord Injury	n=22	43%
Traumatic Brain Injury	n=3	6%
Stroke	n=16	31%
Acquired Brain injury	n=4	8%
Other neurological diseases	n=6	12%
**Education**		
Secondary	n=13	25%

High School Diploma	n=1	2%
Certificate of Professional Standards	n=15	29%
Higher Level Education Cycle	n=7	14%
University Degree	n=15	30%
**Questionnaire form**		
Self-administration	n=24	53%
Interview	n=27	47%
**Assistive Technologies**		
Wheelchair	n=29	
Walking stick	n=8	
Walker	n=3	
Splint	n=7	
Treadmill	n=5	

Reliability Results

The internal consistency reliability of the NATU Quest was analyzed using the Cronbach's Alpha[34] (α=0.895). This result can be interpreted as good reliability.

Reliability through test-retest. A retest was performed 15 days after answering the two questionnaires for the first time to assess the reliability of the NATU Quest. Table 7 shows the weighted quadratic Kappa coefficient and Spearman's coefficient results of the NATU Quest. The results showed a moderate to considerable concordance between NATU Quest items test-retest in the Kappa coefficient because all the results were above 0,50. The results also showed a strong association between test-retest in the Spearman's coefficient (ρ = 0.818), significant with p-value < 0.0001. The results of the ICC showed good reliability (ICC = 0.869; CI 95% 0.781 to 0.923).

Table 7. NATU Quest reliability of the test-retest.

**NATU Quest**				
**Items**		**Weighted Kappa**	**95% Confidence Interval**	
			**Lower Limit**	**Upper Limit**
1	Effectiveness	0.727	0.535	0.918

2	Comfortable	0.733	0.645	0.821
3	Adaptability	0.585	0.434	0.735
4	Easy to put on/off	0.631	0.399	0.863
5	Safe	0.551	0.206	0.895
6	Functionality	0.685	0.541	0.828
7	Ergonomics	0.673	0.507	0.840
8	Easy to use	0.544	0.332	0.755
9	Easy to remember how to use it	0.624	0.298	0.950
10	Satisfaction	0.714	0.557	0.871
**Spearman**		0.818[a]		

[a]The correlation is significant p< 0.0001 (bilateral).

Concurrent validity

The correlation of the total scores between NATU Quest and QUEST 2.0 analyzed with the Spearman's coefficient was strong with ρ = 0.756 significant, with p-value < 0.0001.

Rasch Model Results

All ten items demonstrated a satisfactory fit to the Rasch model, which could be considered productive for measurement (MNSQ infit between 0.64 and 1.43; MNSQ outfit between 0.52 and 1.49).

Discussion

There is a need for a short and easy questionnaire to properly assess the usability of assistive technologies in people with neurological diseases.

The items included in the questionnaire, the format of the questionnaire, and the answer form were derived through two rounds of a Delphi study[27] based on the opinion of 69 experts (neurorehabilitation professionals, such as occupational therapists and

physiotherapists) and 63 users (people with neurological diseases). Finally, we narrowed items down to 10 essential usability items using a content validity ratio. Some of the items also appear in other usability questionnaires. "Effectiveness" and "adaptability" are included in the PIADS [20], while "comfort," "easy to use," "safe," "easy to put on/off," and "satisfaction" appears in the PIADS,[20] the Usability Scale for Assistive Technology for Wheeled Mobility (USAT-WM),[22] and the QUEST 2.0.[23] Items such as "ergonomic" and "functionality" appear in the PIADS[20] and the USAT-WM.[22] The item "easy to remember how to use it" does not appear in any questionnaire but is a usability attribute[15].

Once the questionnaire form was designed, 51 end-users with neurological diseases participated in the questionnaire validity and reliability. The results suggested that NATU Quest has good reliability and validity and fits in the Rasch model.

In contrast with other questionnaires, the NATU Quest was developed, considering the opinions of professionals and people with neurological diseases. Other relevant aspects of this study are the heterogeneity of the included sample, the wide range of neurological diseases, and the inclusion of different assistive technologies.

In this study, we developed a usability scale to analyze assistive technologies for people with neurological diseases; however, the study had some limitations: (1) Selection bias of the participants since most of them were from the same province. However, other regions have the same experiences. [45] (2) Although all the users who participated in the validation had a neurological disease, the authors chose the Pfeiffer test to assess the cognitive problems because it is short and quick to answer. However, Pfeiffer Test does not accurately assess all possible cognitive deficits, and it is not sensitive enough to detect low or mild cognitive deficits. (3) Different assistive technologies were analyzed due to

the people with neurological disease conditions. It would be very interesting to perform another validation with the same product for all users, which may be a good option for developing a new product. (4) Finally, the test-retest reliability was only compared to QUEST 2.0; because we considered that adding other tests for comparison in the same study would have placed a burden on the users.

It would be interesting to measure test-retest reliability with another usability questionnaire for future work. It would also be interesting to verify the new questionnaire's external validity with other population groups, such as older people. Finally, the items should be reviewed after a few years to determine if they are still sensitive enough to assess rapidly evolving assistive technologies. Likewise, it should be interesting to translate the new questionnaire into other languages.

Conclusion

The present study suggested that the NATU is a reliable 10-item usability questionnaire that allows subjective and quick assessment of the usability of assistive technologies. This questionnaire aims to be accessible to people with neurological diseases and reflects the level of acceptance and satisfaction a patient has with the product being used. In addition, the NATU Quest can also be useful for evaluating products in development through user-centered design since the patient can state an opinion about the product during its development, which will facilitate the development of products for a better fit for patients' needs.

Data Availability Statement

All relevant data are within the manuscript and its Supporting Information files.

Conflicts of interest

The authors certify that there is no conflict of interest to declare.

Funding

The author(s) received no specific funding for this work.

Supporting information File

S1_File. NATU Questionnaire

S2_File. All data

Acknowledgments

The authors are grateful to all the professionals and participants involved in this study for their contribution.

Author Contributions

Idea and the project design: Maria Masbernat-Almenara, Eloy Opisso, Esther Duarte-Oller, and Josep Medina-Casanovas.

Data collection: Maria Masbernat-Almenara

Data analysis: Maria Masbernat-Almenara, Eloy Opisso, Francesc Rubí-Carnacea, and Fran Valenzuela-Pascual provided data analysis.

Participants, facilities, and equipment: Josep Medina-Casanovas and Esther Duarte-Oller

Institutional liaisons and consultation: Francesc Rubí-Carnacea and Fran Valenzuela-Pascual

All authors provided writing and revision. All authors read and approved the final version of the manuscript.

References

1. Feigin VL, Nichols E, Alam T, Bannick MS, Beghi E, Blake N, et al. Global, regional, and national burden of neurological disorders, 1990–2016: a systematic analysis for the Global Burden of Disease Study 2016. Lancet Neurol. 2019;18: 459–480. doi:10.1016/S1474-4422(18)30499-X
2. Feigin VL, Krishnamurthi R V., Theadom AM, Abajobir AA, Mishra SR, Ahmed MB, et al. Global, regional, and national burden of neurological disorders during 1990–2015: a systematic analysis for the Global Burden of Disease Study 2015. Lancet Neurol. 2017;16: 877–897. doi:10.1016/S1474-4422(17)30299-5
3. De Mello Monteiro CB, Dawes H, Mayo N, Collett J, Magalhaes FH. Assistive Technology Innovations in Neurological Conditions. Biomed Res Int. 2021;2021. doi:10.1155/2021/6846120
4. Baldassin V, Shimizu HE, Fachin-Martins E. Computer assistive technology and associations with quality of life for individuals with spinal cord injury: a systematic review. Qual Life Res. 2018;27: 597–607. doi:10.1007/S11136-018-1804-9
5. Elnady A, Ben Mortenson W, Menon C. Perceptions of existing wearable robotic devices for upper extremity and suggestions for their development: Findings from therapists and people with stroke. J Med Internet Res. 2018;20: e12. doi:10.2196/rehab.9535
6. Rowland JL, Malone LA, Fidopiastis CM, Padalabalanarayanan S, Thirumalai M, Rimmer JH. Perspectives on Active Video Gaming as a New Frontier in Accessible Physical Activity for Youth With Physical Disabilities. Phys Ther. 2016;96: 521–532. doi:10.2522/PTJ.20140258
7. Sugawara AT, Ramos VD, Alfieri FM, Battistella LR. Abandonment of assistive products: assessing abandonment levels and factors that impact on it. Disabil Rehabil Assist Technol. 2018;13: 716–723. doi:10.1080/17483107.2018.1425748
8. Federici S, Meloni F, Borsci S. The abandonment of assistive technology in Italy: A survey of National Health Service users. Eur J Phys Rehabil Med. 2016;52: 516–526.
9. ISO 9241-11:2018(en), Ergonomics of human-system interaction — Part 11: Usability: Definitions and concepts. [cited October 18 2022]. Available: https://www.iso.org/obp/ui/#iso:std:iso:9241:-11:ed-2:v1:en
10. Almenara M, Cempini M, Gómez C, Cortese M, Martín C, Medina J, et al.

Usability test of a hand exoskeleton for activities of daily living: an example of user-centered design. Disabil Rehabil Assist Technol. 2017;12: 84–96. doi:10.3109/17483107.2015.1079653
11. Nielsen J. Usability engineering. Academic Press; 1993.
12. WHO. Assistive technology. 2018 [cited October 26 2022]. Available: https://www.who.int/news-room/fact-sheets/detail/assistive-technology
13. Friederich A, Bernd T, De Witte L. Methods for the selection of assistive technology in neurological rehabilitation practice. Scand J Occup Ther. 2010;17: 308–318. doi:10.3109/11038120903377082
14. Bernd T, Van Der Pijl D, De Witte LP. Existing models and instruments for the selection of assistive technology in rehabilitation practice. Scand J Occup Ther. 2009;16: 146–158. doi:10.1080/11038120802449362
15. Carneiro L, Rebelo F, Filgueiras E, Noriega P. Usability and User Experience of Technical Aids for People with Disabilities? A Preliminary Study with a Wheelchair. Procedia Manuf. 2015;3: 6068–6074. doi:10.1016/J.PROMFG.2015.07.736
16. Grimm B, Blom A, Jahr H, Rosenbaum D. New Tools and Technologies for Clinical Outcome assessment. J Orthop Transl. 2016;7: 70. doi:10.1016/J.JOT.2016.06.020
17. Brooke john. SUS: A "Quick and Dirty" Usability Scale. 1996; 207–212. doi:10.1201/9781498710411-35
18. Koumpouros Y. A Systematic Review on Existing Measures for the Subjective Assessment of Rehabilitation and Assistive Robot Devices. Journal of Healthcare Engineering. Hindawi Limited; 2016. doi:10.1155/2016/1048964
19. Demers L, Monette M, Descent M, Jutai J, Wolfson C. The psychosocial impact of assistive devices scale (PIADS): Translation and preliminary psychometric evaluation of a Canadian-French version. Qual Life Res. 2002;11: 583–592. doi:10.1023/A:1016397412708
20. Jutai J, Day H. Psychosocial Impact of Assistive Devices Scale (PIADS). Technol Disabil. 2002;14: 107–111. doi:10.3233/tad-2002-14305
21. Scherer MJ, Cushman LA. Measuring subjective quality of life following spinal cord injury: A validation study of the assistive technology device predisposition assessment. Disabil Rehabil. 2001;23: 387–393. doi:10.1080/09638280010006665

22. Arthanat S, Nochajski SM, Lenker JA, Bauer SM, Wu YWB. Measuring usability of assistive Technology from a multicontextual perspective: The case of power wheelchairs. Am J Occup Ther. 2009;63: 751–764. doi:10.5014/ajot.63.6.751

23. Demers L, Weiss-Lambrou R, Ska B. The Quebec User Evaluation of Satisfaction with Assistive Technology (QUEST 2.0): An overview and recent progress. Technol Disabil. 2002;14: 101–105. doi:10.3233/tad-2002-14304

24. Almenara Masbernat M, Medina Casanovas J, Duarte Oller E, Selva O'Callaghan A, Universitat Autònoma de Barcelona. Departament de Medicina. Modelo teórico-práctico para la implementación del diseño centrado en el usuario en el desarrollo, la validación y la aceptación de los productos de apoyo para personas con enfermedades de origen neurológico. 2018.

25. Lavrakas P. Encyclopedia of Survey Research Methods. Encyclopedia of Survey Research Methods. Sage Publications, Inc.; 2012. doi:10.4135/9781412963947

26. Choi YM, Sprigle SH. Approaches for evaluating the usability of assistive technology product prototypes. Assist Technol. 2011;23: 36–41. doi:10.1080/10400435.2010.541407

27. Landeta J. Current validity of the Delphi method in social sciences. Technol Forecast Soc Change. 2006;73: 467–482. doi:10.1016/j.techfore.2005.09.002

28. Tristán-López A. Modificación al modelo de Lawshe para el dictamen cuantitativo de la validez de contenido de un instrumento objetivo. Av en medición. 2008;6: 37–48.

29. Zamanzadeh V, Ghahramanian A, Rassouli M, Abbaszadeh A, Alavi-Majd H, Nikanfar A-R. Design and Implementation Content Validity Study: Development of an instrument for measuring Patient-Centered Communication. J Caring Sci. 2015;4: 165–178. doi:10.15171/jcs.2015.017

30. Jackson JL, Chamberlin J, Kroenke K. Predictors of patient satisfaction. Soc Sci Med. 2001;52: 609–620. doi:10.1016/S0277-9536(00)00164-7

31. Bowling A, Rowe G, Lambert N, Waddington M, Mahtani KR, Kenten C, et al. The measurement of patients' expectations for health care: A review and psychometric testing of a measure of patients' expectations. Health Technology Assessment. 2012. pp. 1–532. doi:10.3310/hta16300

32. Pfeiffer E. A Short Portable Mental Status Questionnaire for the Assessment of Organic Brain Deficit in Elderly Patients. J Am Geriatr Soc. 1975;23: 433–441.

doi:10.1111/j.1532-5415.1975.tb00927.x

33. Martínez de la Iglesia J, Dueñas Herrero R, Onís Vilches MC, Aguado Taberné C, Albert Colomer C, Luque Luque R. [Spanish language adaptation and validation of the Pfeiffer's questionnaire (SPMSQ) to detect cognitive deterioration in people over 65 years of age]. Med Clin (Barc). 2001;117: 129–34. Available: http://www.ncbi.nlm.nih.gov/pubmed/11472684

34. George D, Mallery P. SPSS for Windows step by step : a simple guide and reference, 17.0 update. 4th ed. Boston: Allyn & Bacon, editor. 2003.

35. Streiner DL, Norman GR, Cairney J. Oxford Medicine Online Health Measurement Scales: A practical guide to their development and use (5 ed.). Chapter: Reliability. 2019. doi:10.1093/med/9780199685219.001.0001

36. Marx RG, Menezes A, Horovitz L, Jones EC, Warren RF. A comparison of two time intervals for test-retest reliability of health status instruments. J Clin Epidemiol. 2003;56: 730–735. doi:10.1016/S0895-4356(03)00084-2

37. Fleiss JL, Cohen J. The Equivalence of Weighted Kappa and the Intraclass Correlation Coefficient as Measures of Reliability: http://dx.doi.org/101177/001316447303300309. 2016;33: 613–619. doi:10.1177/001316447303300309

38. Koo TK, Li MY. A Guideline of Selecting and Reporting Intraclass Correlation Coefficients for Reliability Research. J Chiropr Med. 2016;15: 155. doi:10.1016/J.JCM.2016.02.012

39. Mukaka M. A guide to appropriate use of Correlation coefficient in medical research. Malawi Med J. 2012;24: 69. Available: /pmc/articles/PMC3576830/

40. Mora Barrera CA. Validación de la versión en español de la evaluación Quebec de usuarios con tecnologia de asistencia (QUEST 2.0). Universidad Nacional de Colombia. 2010.

41. Boone WJ. Rasch Analysis for Instrument Development: Why, When, and How? CBE Life Sci Educ. 2016;15. doi:10.1187/CBE.16-04-0148

42. Terwee CB, Prinsen CAC, Chiarotto A, Westerman MJ, Patrick DL, Alonso J, et al. COSMIN methodology for evaluating the content validity of patient-reported outcome measures: a Delphi study. Qual Life Res. 2018;27: 1159–1170. doi:10.1007/s11136-018-1829-0

43. Abdul. Quality of Psychology Test Between Likert Scale 5 and 6 Points. J Soc Sci.

2010;6: 399–403. doi:10.3844/jssp.2010.399.403

44. First-person surveys in User Research | by Nikki Anderson | UX Collective. [cited October 27 2022]. Available: https://uxdesign.cc/first-person-surveys-in-user-research-8732c5d9ab96

45. Jiménez-Arberas E, Ordóñez-Fernández FF. Discontinuation or abandonment of mobility assistive technology among people with neurological conditions. Rev Neurol. 2021;72: 426–432. doi:10.33588/RN.7212.2020655

Click here to access/download
Supporting Information
NATU Quest Users Questionnaire - Supporting Information.docx

Supporting Information. NATU QUESTIONNAIRE

Click here to access/download
Supporting Information
S1_File.pdf

Supporting Information. Validity Data

Click here to access/download
Supporting Information
S2_File.xlsx

Developing An Assistive Technology Usability Questionnaire for People with Neurological Diseases

Maria Masbernat-Almenara[1,2,3]; Francesc Rubi-Carnacea [1,2,3*]; Eloy Opisso [4,5,6]; Esther Duarte-Oller [7, 8]; Josep Medina-Casanovas [4,5,6;] Fran Valenzuela-Pascual[1,2,3]

[1] Department of Nursing and Physiotherapy, University of Lleida, Lleida, Spain

[2] Research group of health care. IRB Lleida, Institute for Biomedical Research Dr. Pifarré Foundation, Lleida, Spain

[3] Group on Society Studies, Health, Education and Culture, University of Lleida, Spain

[4] Institut Guttmann, Neurorehabilitation Institute, Badalona, Spain

[5] Universitat Autònoma de Barcelona, Cerdanyola del Vallés, Barcelona, Spain

[6] Fundació Institut d'investigació en Ciències de la Salut Germans Trias i Pujol, Badalona, Barcelona, Spain

[7] Physical Medicine and Rehabilitation Department, Parc de Salut Mar (Hospital del Mar, Hospital de l'Esperança). Barcelona, Catalonia, Spain

[8] Rehabilitation Research Group, Hospital del Mar Medical Research Institute (IMIM). Barcelona, Catalonia, Spain.

*Corresponding Author:

Francesc Rubí Carnacea, Ph.D,

Email: francesc.rubi@udl.cat

Abstract

Purpose. This study describes the development of a questionnaire for assessing the usability of assistive technologies accessible to people with neurological diseases. *Methods.* A Delphi study was conducted to identify relevant items for the questionnaire. After that, the content validity was addressed to identify the essential items. Once the questionnaire was designed following the results of the Delphi study and content validity, the reliability, validity, and the Rasch model of the questionnaire were examined. *Results*. Two rounds of the Delphi study were carried out. A total of 73 participants (42 experts and 31 users) participated in round 1, and 59 people (27 experts and 32 users) in round 2. A total of 53 and 29 items were identified in rounds 1 and 2, respectively. In the content validity, we found nine items above the threshold of 0.58. Finally, ten items were included in the questionnaire. Fifty-one participants participate in the reliability and validity of the questionnaire. The internal consistency reliability of the questionnaire analyzed by Cronbach's Alpha was α=0,895. There was moderate to considerable concordance among our questionnaire items test-retest in the Kappa coefficient and a strong association between test-retest in the Spearman's coefficient ρ=0.818 (p<0,001). The intraclass correlation coefficient was 0,869 with a 95% confidence interval (0,781;0,923). There was a strong correlation between the total scores of the new questionnaire and other validated questionnaires analyzed with Spearman's coefficient ρ=0.756 (p<0,001). The ten items demonstrated a satisfactory fit to the Rasch model. *Conclusions*. The present study suggested that the new questionnaire is a reliable 10-item usability questionnaire that allows subjective and quick assessment of the usability of assistive technologies by people with neurological diseases.

Key words: Nervous System Diseases, Neurological Rehabilitation, Questionnaire Design, Self-Help Devices, User-centered Design

Introduction

Neurological disorders are among the most important causes of disability (247–308 million) and death (8·8–9·4 million) worldwide. [1]In addition, the burden of neurological disorders in public health has increased substantially in the last 25 years because of expanding population numbers, aging, and increased survivor rates from stroke and other neurological disorders.[2] These survivors require intensive rehabilitation to reduce the sequelae of the disorders, increase their quality of life, and improve their autonomy in activities of daily living. In most cases, some assistive technology is needed.

Recent advances in technology and rehabilitation have led to the development of new tools to assess people with disabilities and improve their functionality and autonomy in their daily life.[3] It is known that a good acceptance of assistive technology can improve the quality of life and social inclusion of these patients[4]. However, not all products achieve the goal for which they were designed since they often do not consider the real needs of users.[5] Therefore, it is increasingly necessary to involve end-users from the beginning of developing new products.[6] A product can only be considered successful if it is used, and currently, more than 50% of users abandon their new products because they are not sufficiently usable.[7,8] For this reason, usability is becoming more critical in engineering and rehabilitation. According to the ISO 9241-11 [9], usability is the extent to which specified users can use a product to achieve specified goals with effectiveness, efficiency, and satisfaction in a specified context of use. Understanding this, the design of a product should not involve only multidisciplinary theoretical foundations, but user

experience should be considered throughout the design process to ensure quality, improve usability, and increase product acceptability.[5,10] For that reason, it is essential to perform a usability test during product development to know if it fits the user's needs. Usability testing refers to evaluating a product or service by testing it with representative users [11].

According to the WHO, assistive technology is a general term covering the systems and services related to delivering assistive products and services.[12] Assistive products aim to maintain or improve an individual's functioning and independence, promoting their well-being.[12]

There is a lack of evidence-based procedures for assistive technologies selection [13–15]. For example, professionals prescribe assistive products without considering the user's needs. However, this clinical outcome assessment is important in clinical practice and research because it improves evidence and provides considerable feedback to healthcare professionals and patients, enhancing their empowerment with their opinions and needs and improving their quality of life[16].

Many existing usability questionnaires have been developed to evaluate software usability and web accessibility[17] and not assistive technologies [18]. Some questionnaires[19–21] measure the psychosocial impact of quality of life by using assistive technologies from the point of view of people with disabilities. Those are interesting questionnaires; however, they do not evaluate the usability of assistive technologies per se. There are other questionnaires developed exclusively to assess wheelchairs[22], and only a few are explicitly developed for assistive technologies. For example, the Quebec User Evaluation of Satisfaction with Assistive Technology (QUEST 2.0) [23] contains technical items such as weight, product dimensions, service delivery,

repairs, and device services. People with neurological diseases have limitations in the accessibility of the existing usability questionnaires, for example, in the comprehension of the questions or the answer form. In addition, existing questionnaires are too extensive for them or do not address all the essential items for assessing usability and user satisfaction.[18,24] It is known that longer questionnaires incur high costs in data collection and reduce the number of answers due to the time answering and the quality of the information gathered due to fatigue.[25,26]

The objective of this study was to develop a short questionnaire focused only on the usability of assistive technology products and user satisfaction, and it should be accessible and easy to understand by people with neurological diseases.

Materials and methods

Questionnaire Design

Delphi study

To construct an easy-to-use questionnaire, we performed a Delphi study [27], as it is a prospective approach that seeks to derive a consensus from a group of experts based on the analysis and reflection of a defined problem. A google form questionnaire was sent via email to the neurological healthcare professionals from the private neurorehabilitation hospital of acute neurological patients (Institut Guttmann, Spain) and the public hospital of acute stroke patients (Hospital de l'Esperança, Spain). Also, all the patients from the Institut Guttmann who voluntarily agreed to participate were interviewed to answer the questionnaire.

The questionnaire contains six questions: three were open questions related to assistive technologies, and three were multiple-choice questions about how a questionnaire should be easy and quick to answer. The three open questions were: (1) List some requirements or characteristics important to how the assistive products should or should be manufactured. (2) Would you change anything about the existing assistive products? (3) What kind of assistive products would you like to have? (for users)/ in your workplace? (experts). The three multiple-choice questions related to the questionnaires were: (1) the time required for you to answer a questionnaire, (2) which questionnaire format the user preferred, and (3) how many questions you would like to answer.

Content Validity Ratio

Once the Delphi study finished, and the items were obtained, they were evaluated using the content validity ratio (CVR).[28,29] Content validity addresses the degree to which items of an instrument sufficiently represent the content domain and answers the question of to what extent the selected sample in an instrument or instrument items is a comprehensive sample of the content. [29] The content validity ratio varies between 1 and -1. Higher scores indicate greater agreement by panel members on the need for an item in an instrument. The formula of content validity ratio is CVR= (Ne - N/2)/(N/2), in which the Ne is the number of panelists indicating "essential" and N is the total number of panelists.[29] The numeric value of the content validity ratio is determined by Lawshe Table.[28]

Questionnaire design

The questions were written based on the qualitative data obtained in the Delphi study and the items obtained in the CVR. Subsequently, the users (people with neurological

diseases) read the questionnaire to assess their understanding of the questions and if their answers could be represented with the proposed response values (numbers, traffic light colors, and faces).

Questionnaire reliability and validity

The reliability and validity of the new questionnaire were addressed.

Sample size. The recommended sample size for similar studies has been established as 50 subjects.[30,31] The inclusion criteria for participants were (1) adults (≥18 years old) with neurological diseases that have been using some assistive products for the past month. Exclusion criteria moderate to severe cognitive impairment based on the Pfeiffer SPMSQ test [32] translated into Spanish[33].

Assistive product analyzed: It is challenging to analyze the same product because neurological patients have personalized assistive technologies adapted to their disability. Therefore, all the participants could choose which product addressed, only with the condition that they had used it for at least one month.

Data analysis of Reliability

The reliability and validity of the new questionnaire and all the data were analyzed using the SPSS Statistics 27 program.

The internal consistency reliability of the new questionnaire was analyzed using Cronbach's Alpha.[34] The value of the coefficients was interpreted as follows: unacceptable ($0.5 \geq \alpha$), poor ($0.6 \geq \alpha \geq 0.5$), questionable ($0.7 \geq \alpha \geq 0.6$), acceptable ($0.8 \geq \alpha \geq 0.7$). good ($0.9 \geq \alpha \geq 0.8$), and excellent $\alpha \geq 0.9$).[34]

Test-retest. Reliability can be assessed with a test-retest comparison. This method evaluates the stability of the results at two different points in time using a stability coefficient. To perform the test-retest, the same questionnaire was administered on two occasions, separated by a certain period, to the same subjects and under the same conditions. According to different authors,[35,36] an interval between two days and two weeks between the test-retest interviews is recommended. Accordingly, the interval between the two tests was set to 15 days. In order to know the concordance between the test and the retest, data was analyzed by weighted quadratic Kappa coefficient. [37] The value of the coefficients was interpreted as follows: poor (< 0.00), weak (0.00–0.20), good (0.20–0.40), moderate (0.41–0.60), considerable (0.61–0.80), and almost perfect (0.81–1.00).[37]

Intraclass correlation coefficient (ICC) model 1.1 with a 95% of confidence interval (CI) was measured. ICC indicates the degree to which the participants maintained their opinion during the test-retest.[38] The value of the coefficients was interpreted as follows: poor reliability (< 0.5), moderate reliability (0.5–0.75), good reliability (0.75–0.9), and excellent reliability (> 0.90).[38]

The normality of the data was tested using the Kolmogorov-Smirnov test, and the repeatability was determined by the Spearman's correlation coefficient[39] between the test and the retest.

Data Analysis of validity

Concurrent validity. To know the relationship between the new questionnaire and another related questionnaire, QUEST 2.0[23] was chosen. It is the shortest worldwide questionnaire and has been translated and validated in several languages. Only eight items

of the QUEST 2.0 was administered to users in Spanish[40] because the other four items are related to services. Also, in this study, data was tested using the Kolmogorov-Smirnov test and analyzed using Spearman's correlation coefficient [39].

Measurement of other parameters. The time was measured while the participants answered the questionnaire to obtain the average time of all the participants.

Rasch Model

In order to further analyze the construct validity of the different items, the Rasch model [41] was carried out. This model gives an idea of the scale's internal consistency by relating the item's difficulty to the person's ability[41]. The values of infit and outfit mean squares (MNSQ) of 1 indicate a perfect fit between the data and the model, values between 0.5 and 1.5 indicate an acceptable fit, and values greater than 2 indicate a severe mismatch[41].

The study was developed following the COSMIN guidelines[42].

Ethical Approval

The Institut Guttmann Neurorehabilitation Hospital Ethics Committee approved this study. In addition, this research was conducted following the Declaration of Helsinki's ethical principles. All participants participated voluntarily, and they signed an informed written consent form. Their personal data were archived following the Spanish Organic Law 3/2018, December 5, on the Protection of Personal Data and guarantee of digital rights.

Results

Study Delphi

Two rounds of a Delphi study were needed to obtain all the items of the new questionnaire. A total of 73 participants (42 experts and 31 users) were involved in round 1 (Table 1). From round 1, 53 different items and qualitative information about questionnaires were derived qualitatively.

Round two involved 59 people (27 experts and 32 users) (Table 1). This round obtained 15 items and data about the scale. The items were: "effectiveness", "comfort", "adaptability", "easy to put on/off", "safe", "lightweight", "functionality", "ergonomic", "economical", "affordable", "easy to use", "feedback", "stimulating," "monitored" and "movement facilitator. Due to "economical" and "affordable" have the same meaning, both were considered as a single item. Additionally, in this round and following the usability premises, the items "aesthetics" and "easy to remember how to use it" were added. Finally, 16 items were analyzed using the content validity ratio.

Table 1. Demographic Characteristics of participants of the two rounds in the Delphi Study.

EXPERT PANEL	Round 1 n=42		Round 2 n= 27[a]	
**Demographic Characteristics**	**Number**	**Percentage**	**Number**	**Percentage**
**Age**				
Between 18 and 25 years	n= 3	7%	n=1	4%
Between 26 and 35 years	n=17	41%	n=11	41%
Between 36 and 45 years	n=11	26%	n=8	29%
Between 46 and 65 years	n= 11	26%	n=7	26%
More than 65 years	n= 0	0%	n=0	0%
**Sex**				
Female	N=30	71%	19	70%

Male	N=12	29%	8	30%
**Profession**				
Physician	n=9	21%	6	22%
Physiotherapist	n=14	33%	9	33%
Occupational Therapist	n=13	31%	8	30%
Other[b]	n=6	15%	4	15%
**Expert in Neurorehabilitation**				
Yes	39	93%	27	100%
**USER PANEL**	**Round 1 n= 31**		**Round 2 n= 32[d]**	
**Demographic Characteristics**	**Number**	**Percentage**	**Number**	**Percentage**
**Age**				
Between 18 and 25 years	n= 4	13%	n=1	3%
Between 26 and 35 years	n=6	19%	n=9	28%
Between 36 and 45 years	n=5	16%	n=3	10%
Between 46 and 65 years	n= 13	42%	n=16	50%
More than 65 years	n= 3	10%	n=3	9%
**Sex**				
Female	n=17	45%	n=16	50%
Male	n=14	55%	n=16	50%
**Pathology**				
Spinal Cord Injury	n=13	42%	n=7	22%
Traumatic Brain Injury	n=5	16%	n=1	3%
Stroke	n=8	26%	n=13	41%
Other	n=5	16%	n=11	34%
**Assistive Technologies**				
Wheelchair	n= 23	53%	n=19	36%
Canes	n= 8	18%	n=12	23%
Orthesis	n=8	19%	n=15	28%
Other	n=2	5%	n=6	11%
Nothing[c]	n=2	5%	n=1	2%

[a]In the second round, 36% did not answer the questionnaire. [b]Others are, for example, orthopedic professionals and engineers. [c]Nothing means that he/she did not use assistive technologies at the moment of the interview; however, they used them in the past. [d]Users are different from round 1.

Content Validity Ratio (CVR)

Thirty-four experts from the Delphi study from the private neurorehabilitation hospital of acute neurological patients and the public hospital of acute stroke patients were selected to evaluate the items obtained from round two. 70% of the participants have more than ten years of expertise in neurorehabilitation, and all of them used to work with assistive technologies experts (Table 2).

Table 2. Demographic Characteristics of participants in Content Validity.

	**Content Validity n=34**	
**Demographic Characteristics**	**Number**	**Percentage**
**Age**		
Between 26 and 35 years	n=8	24%
Between 36 and 45 years	n=12	35%
Between 46 and 65 years	n= 14	41%
**Sex**		
Female	N=21	62%
Male	N=13	38%
**Profession**		
Physician	n= 11	32%
Physiotherapist	n=14	41%
Occupational Therapist	n=7	21%
Other[a]	n=2	6%
**Expert in Neurorehabilitation[b]**		
Between 1 and 5 years	n=5	15%
Between 5 and 10 years	n=5	15%
Between 10 and 20 years	n=12	35%
More than 20 years	n=12	35%

[a]Others are, for example, orthopedic professionals and engineers. [b]All the participants used to work with people with neurological diseases.

Participants had to choose which items were *essential*, *useful but not essential*, and *not essential* when evaluating the items. Table 3 shows the results. Nine of the sixteen items exceeded the threshold of 0.58. In addition, the experts agreed to accept the item "comfortable" because its threshold was 0.53.

Table 3. Content Validity Ratio results.

Items	ne	N	CVR[a]
1. Effectiveness	33	34	0,97
2. Comfortable	18	34	0,53
3. Adaptability	21	34	0,62
4. Easy to put on/off	20	34	0,59
5. Safe	32	34	0,94
6. Lightweight	12	34	0,35
7. Functionality	31	34	0,91
8. Ergonomic	22	34	0,65
9. Economical/ Affordable	13	34	0,38
10. Easy to use	24	34	0,71
11. Aesthetics	4	34	0,12
12. Easy to remember how to use it	20	34	0,59
13. Feedback	16	34	0,47
14. Stimulating	14	34	0,41
15. Monitored	10	34	0,29
16. Movement Facilitator	21	34	0,62

[a]CVR (content validity ratio) = (Ne-N/2) / (N/2) with 34 people in the expert panel (N=34), the items with a CVR bigger than 0.58 (in bold) will remain at the instrument, and the rest will be eliminated.

The experts considered "functional" and "movement facilitator" as one word due to the similarity of their meanings. The item "satisfaction" was added to the questionnaire to ascertain the end-user opinion of the product. Therefore, ten items were selected to create the questionnaire (Table 4).

Table 4. Questions and the items that are involved in each question.

Questions	Items

1	I believe that _____ can help me improve my functional independence	Effectiveness
2	I feel comfortable wearing/using ____	Comfortable
3	_____ adapts to my characteristics and needs	Adaptability
4	Donning/Doffing …………….. is quick and easy for me.	Easy to put on / off
5	I feel safe using/wearing _____ / _____ is safe in its use	Safe
6	_____ allows me to achieve my goal/ allows me to perform a movement/action that I could not do before	Functionality/ movement facilitator
7	_____ adapts to my daily live needs	Ergonomics
8	In general, _____ is easy to use	Easy to use
9	Information and instructions of use______ are easy to understand and easy to remember	Easy to remember how to use it
10	Overall, I am satisfied with______	Satisfaction

Questionnaire design

Following the qualitative information from the Delphi study, the questionnaire was formulated in an understandable language, and the length was as short as possible. When necessary, the users could fill the blank space with the product's name being evaluated. The 6-point Likert scale was chosen since it forces the respondent to decide positively or negatively according to the item in question[43]. A numeric panel from 0 to 5 points was used, and each number was associated with a box using the colors of the traffic light to facilitate the choice. Furthermore, faces with expressions were added to facilitate the answers according to the patients with neurological diseases responses from the Delphi study.

The statements of the questions are in first person to facilitate users' answers and usability experiences of the product subjectively [44].

Once the questionnaire was finished, ten users (see demographic characteristics in table 5) read and answered the questionnaire to know if all the requirements from the Delphi study were met. During this process, some wording modifications were made.

Table 5. Demographic characteristics users from the questionnaire design.

Questionnaire analysis	n= 10	
**Demographic Characteristics**	**Number**	**Percentage**
**Age**		
Between 18 and 25 years	n=1	10%
Between 26 and 35 years	n=2	20%
Between 36 and 45 years	n=2	20%
Between 46 and 65 years	n= 3	30%
More than 65 years	n= 2	20%
**Sex**		
Female	n=6	60%
Male	n=4	40%
**Pathology**		
Spinal Cord Injury	n=4	40%
Traumatic Brain Injury	n= 2	20%
Stroke	n= 3	30%
Other	n= 1	10%
**Assistive Technologies**		
Wheelchair	n= 8	80%
Canes	n= 1	10%
Orthesis	n=4	40%
Other	n=1	10%
Nothing[a]	n=1	10%

[a]Nothing means that he/she did not use assistive technologies at the moment of the interview; however, they used it in the past

The new questionnaire was named "Assistive Technology Usability Questionnaire for people with Neurological diseases" (NATU Quest) and included ten questions. It should be administered at the end of a usability test. The final version of the questionnaire, questionnaire score, and interpretation are available in the supporting information file.

Reliability and Validity

Sample description

A total of 51 people with neurological diseases consecutive recruited from the Institut Guttmann Hospital voluntarily agreed to participate in the study. These people were different from the Delphi study, and their demographic characteristics are summarized in Table 6. Fifty-three percent of the participants answered the questionnaire through an interview due to physical limitations, and the rest (47%) answered it by themselves. First, the participants answered the new questionnaire and the QUEST 2.0. On average, the participants completed the new questionnaire within 102.40 seconds in the first administration and within 82.08 seconds in the second administration. The QUEST 2.0 was administered once before the NATU Quest, and the participants needed an average of 74 seconds to complete it. Participants scored an assistive product they had used in the last three months. All the patients answered all the items.

Table 6. Demographic characteristics of the 51 participants in the reliability and validity of NATU Quest.

**Demographic Characteristics**	**Mean /Number**	**Percentage**
Age, range 16-72 years	Mean = 48	
**Sex**		
Female	n=20	39%
Male	n= 31	61%
**Pathology**		
Spinal cord Injury	n=22	43%
Traumatic Brain Injury	n=3	6%
Stroke	n=16	31%
Acquired Brain injury	n=4	8%
Other neurological diseases	n=6	12%
**Education**		
Secondary	n=13	25%

High School Diploma	n=1	2%
Certificate of Professional Standards	n=15	29%
Higher Level Education Cycle	n=7	14%
University Degree	n=15	30%
**Questionnaire form**		
Self-administration	n=24	53%
Interview	n=27	47%
**Assistive Technologies**		
Wheelchair	n=29	
Walking stick	n=8	
Walker	n=3	
Splint	n=7	
Treadmill	n=5	

Reliability Results

The internal consistency reliability of the NATU Quest was analyzed using the Cronbach's Alpha[34] (α=0.895). This result can be interpreted as good reliability.

Reliability through test-retest. A retest was performed 15 days after answering the two questionnaires for the first time to assess the reliability of the NATU Quest. Table 7 shows the weighted quadratic Kappa coefficient and Spearman's coefficient results of the NATU Quest. The results showed a moderate to considerable concordance between NATU Quest items test-retest in the Kappa coefficient because all the results were above 0,50. The results also showed a strong association between test-retest in the Spearman's coefficient (ρ = 0.818), significant with p-value < 0.0001. The results of the ICC showed good reliability (ICC = 0.869; CI 95% 0.781 to 0.923).

Table 7. NATU Quest reliability of the test-retest.

**NATU Quest**				
**Items**		**Weighted Kappa**	**95% Confidence Interval**	
			**Lower Limit**	**Upper Limit**
1	Effectiveness	0.727	0.535	0.918

2	Comfortable	0.733	0.645	0.821
3	Adaptability	0.585	0.434	0.735
4	Easy to put on/off	0.631	0.399	0.863
5	Safe	0.551	0.206	0.895
6	Functionality	0.685	0.541	0.828
7	Ergonomics	0.673	0.507	0.840
8	Easy to use	0.544	0.332	0.755
9	Easy to remember how to use it	0.624	0.298	0.950
10	Satisfaction	0.714	0.557	0.871
**Spearman**		0.818[a]		

[a]The correlation is significant p< 0.0001 (bilateral).

Concurrent validity

The correlation of the total scores between NATU Quest and QUEST 2.0 analyzed with the Spearman's coefficient was strong with $\rho = 0.756$ significant, with p-value < 0.0001.

Rasch Model Results

All ten items demonstrated a satisfactory fit to the Rasch model, which could be considered productive for measurement (MNSQ infit between 0.64 and 1.43; MNSQ outfit between 0.52 and 1.49).

Discussion

There is a need for a short and easy questionnaire to properly assess the usability of assistive technologies in people with neurological diseases.

The items included in the questionnaire, the format of the questionnaire, and the answer form were derived through two rounds of a Delphi study[27] based on the opinion of 69 experts (neurorehabilitation professionals, such as occupational therapists and

physiotherapists) and 63 users (people with neurological diseases). Finally, we narrowed items down to 10 essential usability items using a content validity ratio. Some of the items also appear in other usability questionnaires. "Effectiveness" and "adaptability" are included in the PIADS [20], while "comfort," "easy to use," "safe," "easy to put on/off," and "satisfaction" appears in the PIADS,[20] the Usability Scale for Assistive Technology for Wheeled Mobility (USAT-WM),[22] and the QUEST 2.0.[23] Items such as "ergonomic" and "functionality" appear in the PIADS[20] and the USAT-WM.[22] The item "easy to remember how to use it" does not appear in any questionnaire but is a usability attribute[15].

Once the questionnaire form was designed, 51 end-users with neurological diseases participated in the questionnaire validity and reliability. The results suggested that NATU Quest has good reliability and validity and fits in the Rasch model.

In contrast with other questionnaires, the NATU Quest was developed, considering the opinions of professionals and people with neurological diseases. Other relevant aspects of this study are the heterogeneity of the included sample, the wide range of neurological diseases, and the inclusion of different assistive technologies.

In this study, we developed a usability scale to analyze assistive technologies for people with neurological diseases; however, the study had some limitations: (1) Selection bias of the participants since most of them were from the same province. However, other regions have the same experiences. [45] (2) Although all the users who participated in the validation had a neurological disease, the authors chose the Pfeiffer test to assess the cognitive problems because it is short and quick to answer. However, Pfeiffer Test does not accurately assess all possible cognitive deficits, and it is not sensitive enough to detect low or mild cognitive deficits. (3) Different assistive technologies were analyzed due to

the people with neurological disease conditions. It would be very interesting to perform another validation with the same product for all users, which may be a good option for developing a new product. (4) Finally, the test-retest reliability was only compared to QUEST 2.0; because we considered that adding other tests for comparison in the same study would have placed a burden on the users.

It would be interesting to measure test-retest reliability with another usability questionnaire for future work. It would also be interesting to verify the new questionnaire's external validity with other population groups, such as older people. Finally, the items should be reviewed after a few years to determine if they are still sensitive enough to assess rapidly evolving assistive technologies. Likewise, it should be interesting to translate the new questionnaire into other languages.

Conclusion

The present study suggested that the NATU is a reliable 10-item usability questionnaire that allows subjective and quick assessment of the usability of assistive technologies. This questionnaire aims to be accessible to people with neurological diseases and reflects the level of acceptance and satisfaction a patient has with the product being used. In addition, the NATU Quest can also be useful for evaluating products in development through user-centered design since the patient can state an opinion about the product during its development, which will facilitate the development of products for a better fit for patients' needs.

Data Availability Statement

All relevant data are within the manuscript and its Supporting Information files.

Conflicts of interest

The authors certify that there is no conflict of interest to declare.

Funding

The author(s) received no specific funding for this work.

Supporting information File

S1_File. NATU Questionnaire

S2_File. All data

Acknowledgments

The authors are grateful to all the professionals and participants involved in this study for their contribution.

Author Contributions

Idea and the project design: Maria Masbernat-Almenara, Eloy Opisso, Esther Duarte-Oller, and Josep Medina-Casanovas.

Data collection: Maria Masbernat-Almenara

Data analysis: Maria Masbernat-Almenara, Eloy Opisso, Francesc Rubí-Carnacea, and Fran Valenzuela-Pascual provided data analysis.

Participants, facilities, and equipment: Josep Medina-Casanovas and Esther Duarte-Oller

Institutional liaisons and consultation: Francesc Rubí-Carnacea and Fran Valenzuela-Pascual

All authors provided writing and revision. All authors read and approved the final version of the manuscript.

References

1. Feigin VL, Nichols E, Alam T, Bannick MS, Beghi E, Blake N, et al. Global, regional, and national burden of neurological disorders, 1990–2016: a systematic analysis for the Global Burden of Disease Study 2016. Lancet Neurol. 2019;18: 459–480. doi:10.1016/S1474-4422(18)30499-X
2. Feigin VL, Krishnamurthi R V., Theadom AM, Abajobir AA, Mishra SR, Ahmed MB, et al. Global, regional, and national burden of neurological disorders during 1990–2015: a systematic analysis for the Global Burden of Disease Study 2015. Lancet Neurol. 2017;16: 877–897. doi:10.1016/S1474-4422(17)30299-5
3. De Mello Monteiro CB, Dawes H, Mayo N, Collett J, Magalhaes FH. Assistive Technology Innovations in Neurological Conditions. Biomed Res Int. 2021;2021. doi:10.1155/2021/6846120
4. Baldassin V, Shimizu HE, Fachin-Martins E. Computer assistive technology and associations with quality of life for individuals with spinal cord injury: a systematic review. Qual Life Res. 2018;27: 597–607. doi:10.1007/S11136-018-1804-9
5. Elnady A, Ben Mortenson W, Menon C. Perceptions of existing wearable robotic devices for upper extremity and suggestions for their development: Findings from therapists and people with stroke. J Med Internet Res. 2018;20: e12. doi:10.2196/rehab.9535
6. Rowland JL, Malone LA, Fidopiastis CM, Padalabalanarayanan S, Thirumalai M, Rimmer JH. Perspectives on Active Video Gaming as a New Frontier in Accessible Physical Activity for Youth With Physical Disabilities. Phys Ther. 2016;96: 521–532. doi:10.2522/PTJ.20140258
7. Sugawara AT, Ramos VD, Alfieri FM, Battistella LR. Abandonment of assistive products: assessing abandonment levels and factors that impact on it. Disabil Rehabil Assist Technol. 2018;13: 716–723. doi:10.1080/17483107.2018.1425748
8. Federici S, Meloni F, Borsci S. The abandonment of assistive technology in Italy: A survey of National Health Service users. Eur J Phys Rehabil Med. 2016;52: 516–526.
9. ISO 9241-11:2018(en), Ergonomics of human-system interaction — Part 11: Usability: Definitions and concepts. [cited October 18 2022]. Available: https://www.iso.org/obp/ui/#iso:std:iso:9241:-11:ed-2:v1:en
10. Almenara M, Cempini M, Gómez C, Cortese M, Martín C, Medina J, et al.

Usability test of a hand exoskeleton for activities of daily living: an example of user-centered design. Disabil Rehabil Assist Technol. 2017;12: 84–96. doi:10.3109/17483107.2015.1079653

11. Nielsen J. Usability engineering. Academic Press; 1993.
12. WHO. Assistive technology. 2018 [cited October 26 2022]. Available: https://www.who.int/news-room/fact-sheets/detail/assistive-technology
13. Friederich A, Bernd T, De Witte L. Methods for the selection of assistive technology in neurological rehabilitation practice. Scand J Occup Ther. 2010;17: 308–318. doi:10.3109/11038120903377082
14. Bernd T, Van Der Pijl D, De Witte LP. Existing models and instruments for the selection of assistive technology in rehabilitation practice. Scand J Occup Ther. 2009;16: 146–158. doi:10.1080/11038120802449362
15. Carneiro L, Rebelo F, Filgueiras E, Noriega P. Usability and User Experience of Technical Aids for People with Disabilities? A Preliminary Study with a Wheelchair. Procedia Manuf. 2015;3: 6068–6074. doi:10.1016/J.PROMFG.2015.07.736
16. Grimm B, Blom A, Jahr H, Rosenbaum D. New Tools and Technologies for Clinical Outcome assessment. J Orthop Transl. 2016;7: 70. doi:10.1016/J.JOT.2016.06.020
17. Brooke john. SUS: A "Quick and Dirty" Usability Scale. 1996; 207–212. doi:10.1201/9781498710411-35
18. Koumpouros Y. A Systematic Review on Existing Measures for the Subjective Assessment of Rehabilitation and Assistive Robot Devices. Journal of Healthcare Engineering. Hindawi Limited; 2016. doi:10.1155/2016/1048964
19. Demers L, Monette M, Descent M, Jutai J, Wolfson C. The psychosocial impact of assistive devices scale (PIADS): Translation and preliminary psychometric evaluation of a Canadian-French version. Qual Life Res. 2002;11: 583–592. doi:10.1023/A:1016397412708
20. Jutai J, Day H. Psychosocial Impact of Assistive Devices Scale (PIADS). Technol Disabil. 2002;14: 107–111. doi:10.3233/tad-2002-14305
21. Scherer MJ, Cushman LA. Measuring subjective quality of life following spinal cord injury: A validation study of the assistive technology device predisposition assessment. Disabil Rehabil. 2001;23: 387–393. doi:10.1080/09638280010006665

22. Arthanat S, Nochajski SM, Lenker JA, Bauer SM, Wu YWB. Measuring usability of assistive Technology from a multicontextual perspective: The case of power wheelchairs. Am J Occup Ther. 2009;63: 751–764. doi:10.5014/ajot.63.6.751

23. Demers L, Weiss-Lambrou R, Ska B. The Quebec User Evaluation of Satisfaction with Assistive Technology (QUEST 2.0): An overview and recent progress. Technol Disabil. 2002;14: 101–105. doi:10.3233/tad-2002-14304

24. Almenara Masbernat M, Medina Casanovas J, Duarte Oller E, Selva O'Callaghan A, Universitat Autònoma de Barcelona. Departament de Medicina. Modelo teórico-práctico para la implementación del diseño centrado en el usuario en el desarrollo, la validación y la aceptación de los productos de apoyo para personas con enfermedades de origen neurológico. 2018.

25. Lavrakas P. Encyclopedia of Survey Research Methods. Encyclopedia of Survey Research Methods. Sage Publications, Inc.; 2012. doi:10.4135/9781412963947

26. Choi YM, Sprigle SH. Approaches for evaluating the usability of assistive technology product prototypes. Assist Technol. 2011;23: 36–41. doi:10.1080/10400435.2010.541407

27. Landeta J. Current validity of the Delphi method in social sciences. Technol Forecast Soc Change. 2006;73: 467–482. doi:10.1016/j.techfore.2005.09.002

28. Tristán-López A. Modificación al modelo de Lawshe para el dictamen cuantitativo de la validez de contenido de un instrumento objetivo. Av en medición. 2008;6: 37–48.

29. Zamanzadeh V, Ghahramanian A, Rassouli M, Abbaszadeh A, Alavi-Majd H, Nikanfar A-R. Design and Implementation Content Validity Study: Development of an instrument for measuring Patient-Centered Communication. J Caring Sci. 2015;4: 165–178. doi:10.15171/jcs.2015.017

30. Jackson JL, Chamberlin J, Kroenke K. Predictors of patient satisfaction. Soc Sci Med. 2001;52: 609–620. doi:10.1016/S0277-9536(00)00164-7

31. Bowling A, Rowe G, Lambert N, Waddington M, Mahtani KR, Kenten C, et al. The measurement of patients' expectations for health care: A review and psychometric testing of a measure of patients' expectations. Health Technology Assessment. 2012. pp. 1–532. doi:10.3310/hta16300

32. Pfeiffer E. A Short Portable Mental Status Questionnaire for the Assessment of Organic Brain Deficit in Elderly Patients. J Am Geriatr Soc. 1975;23: 433–441.

doi:10.1111/j.1532-5415.1975.tb00927.x
33. Martínez de la Iglesia J, Dueñas Herrero R, Onís Vilches MC, Aguado Taberné C, Albert Colomer C, Luque Luque R. [Spanish language adaptation and validation of the Pfeiffer's questionnaire (SPMSQ) to detect cognitive deterioration in people over 65 years of age]. Med Clin (Barc). 2001;117: 129–34. Available: http://www.ncbi.nlm.nih.gov/pubmed/11472684
34. George D, Mallery P. SPSS for Windows step by step : a simple guide and reference, 17.0 update. 4th ed. Boston: Allyn & Bacon, editor. 2003.
35. Streiner DL, Norman GR, Cairney J. Oxford Medicine Online Health Measurement Scales: A practical guide to their development and use (5 ed.). Chapter: Reliability. 2019. doi:10.1093/med/9780199685219.001.0001
36. Marx RG, Menezes A, Horovitz L, Jones EC, Warren RF. A comparison of two time intervals for test-retest reliability of health status instruments. J Clin Epidemiol. 2003;56: 730–735. doi:10.1016/S0895-4356(03)00084-2
37. Fleiss JL, Cohen J. The Equivalence of Weighted Kappa and the Intraclass Correlation Coefficient as Measures of Reliability: http://dx.doi.org/101177/001316447303300309. 2016;33: 613–619. doi:10.1177/001316447303300309
38. Koo TK, Li MY. A Guideline of Selecting and Reporting Intraclass Correlation Coefficients for Reliability Research. J Chiropr Med. 2016;15: 155. doi:10.1016/J.JCM.2016.02.012
39. Mukaka M. A guide to appropriate use of Correlation coefficient in medical research. Malawi Med J. 2012;24: 69. Available: /pmc/articles/PMC3576830/
40. Mora Barrera CA. Validación de la versión en español de la evaluación Quebec de usuarios con tecnologia de asistencia (QUEST 2.0). Universidad Nacional de Colombia. 2010.
41. Boone WJ. Rasch Analysis for Instrument Development: Why, When, and How? CBE Life Sci Educ. 2016;15. doi:10.1187/CBE.16-04-0148
42. Terwee CB, Prinsen CAC, Chiarotto A, Westerman MJ, Patrick DL, Alonso J, et al. COSMIN methodology for evaluating the content validity of patient-reported outcome measures: a Delphi study. Qual Life Res. 2018;27: 1159–1170. doi:10.1007/s11136-018-1829-0
43. Abdul. Quality of Psychology Test Between Likert Scale 5 and 6 Points. J Soc Sci.

2010;6: 399–403. doi:10.3844/jssp.2010.399.403

44. First-person surveys in User Research | by Nikki Anderson | UX Collective. [cited October 27 2022]. Available: https://uxdesign.cc/first-person-surveys-in-user-research-8732c5d9ab96

45. Jiménez-Arberas E, Ordóñez-Fernández FF. Discontinuation or abandonment of mobility assistive technology among people with neurological conditions. Rev Neurol. 2021;72: 426–432. doi:10.33588/RN.7212.2020655

Dear Editor of PLOS ONE,

I am pleased to resubmit the revised version of PONE-D-22-13676 " Developing An Assistive Technology Usability Questionnaire for People with Neurological Diseases" for your consideration. We are very grateful for the excellent suggestions and comments from the reviewers. As you suggested, we have carefully considered all the suggestions and comments and made revisions accordingly. Thanks again for those valuable suggestions, which have helped us further improve the quality and clarity of this manuscript. We have addressed each reviewer's requirements as outlined below, which are indicated in the manuscript in yellow.

Editor's Comments to Author:

1. Please ensure that your manuscript meets PLOS ONE's style requirements, including those for file naming. The PLOS ONE style templates can be found at

https://journals.plos.org/plosone/s/file?id=wjVg/PLOSOne_formatting_sample_main_body.pdf and https://journals.plos.org/plosone/s/file?id=ba62/PLOSOne_formatting_sample_title_authors_affiliations.pdf.

We appreciate the reviewer's comment. The paper has been carefully revised to meet PLOS ONE's style requirements. Also, the manuscript has been edited by an English-speaking native.

2. Please provide additional details regarding participant consent. In the ethics statement in the Methods and online submission information, please ensure that you have specified what type you obtained (for instance, written or verbal, and if verbal, how it was documented and witnessed). If your study included minors, state whether you obtained consent from parents or guardians. If the need for consent was waived by the ethics committee, please include this information.

We appreciate the editor's suggestion. We have added "written" to clarify this point. Please see page 9, line 201, and below:

All participants participated voluntarily, and they signed an informed written consent form.

3. In your Data Availability statement, you have not specified where the minimal data set underlying the results described in your manuscript can be found. PLOS defines a study's minimal data set as the underlying data used to reach the conclusions drawn in the

manuscript and any additional data required to replicate the reported study findings in their entirety. All PLOS journals require that the minimal data set be made fully available. For more information about our data policy, please see http://journals.plos.org/plosone/s/data-availability.

Following your advice, we have added a Data availability statement. Please see page 20, line 372, and below:

Data Availability Statement

All relevant data are within the manuscript and its Supporting Information files.

Also, we have uploaded a new supporting information file with reliability and validity data.

REVIEWER 1

1. Assistive Technologies, instead of Nervous System Diseases????

We thank the reviewer's observation. MESH term of Assistive Technologies is Self-Help Devices.

2. functioning, instead

We thank the reviewer's observation. However, we want to express functionality instead of functioning.

3. This definition is very questionable! The authors have taken and written ipsis verbis from [10] who, in turn, took it from Fed Regist. 1991;56(60):4121... 1991!!!!!!!!!!!!!!!!! It will be useful to reformulate this text and, consequently, the conceptual framework of this definition.

According to the reviewer, we have added a new definition. Please see page 4, line 76 and below.

According to the WHO, assistive technology is a general term covering the systems and services related to delivering assistive products and services.[12] Assistive products aim to maintain or improve an individual's functioning and independence, promoting their well-being.[12]

4. To use the term "person with neurological diseases" is not entirely correct. I think the authors want to develop a tool to assess the usability of assistive technology products and user satisfaction and make it accessible to everyone, including those with communication limitations/restritions, cognitive/mental deficits/etc. It would not be appropriate to talk about "neurological disorders" in general, as there are some persons with neurological diseases, but who do not present any difficulty in understanding and answering the existing questionnaires.

We appreciate the reviewer's comment and agree; however, this questionnaire was developed and tested by people with neurological diseases. It is right that some people do not present mental/cognitive disorders, and the questionnaire will be easier for them.

5. Why did you use the "Short Portable Mental Status Questionnaire for the Assessment of Organic Brain Deficit in Elderly Patients", since it is an instrument for the elderly population and the sample used ranged from 16 to 72 years (mean age 48 years)?

Thank you for your valuable observation. We agree with the reviewer's comment, and we chose the Pfeiffer test instead of MMSE because it is shorter, and we thought that should be too many questionnaires at the same time (Pfeiffer, Quest 2.0, and the new questionnaire).

6.Don't you think it is too short??!! Aren't the users still learning how to dela with the assistive device?

We thank the reviewer for this suggestion. We chose at least one month because we performed the study in an acute and subacute hospital. People usually stay in the hospital for between 3-6 months, depending on the severity of the pathology and if they are in a private or public

insurance. When the people are autonomous, they are discharged. We choose only one month to perform the pre and the post before being discharged.

7. Explanation?

We appreciate the reviewer's comment. These patients did not use the AT at the moment of the interview; however, they used it in the past (last weeks, months)
We have added a sentence to clarify it. Please see table 1, pages 10 and 11, and below:

[c]Nothing means that he/she did not use assistive technologies at the moment of the interview; however, they used them in the past.

8. Why from the acute?!

We appreciate the reviewer's suggestion. The hospitals where we performed the study were the Institut Guttmann (Badalona) and the Hospital de l'Esperança (Barcelona) in Spain; both specialized in acute and subacute patients.

9. Do you search for references/support? Please use a Reference.

Following the reviewer's suggestion, we have added a new reference. Please see page 14, line 270.

The statements of the questions are in first person to facilitate users' answers and usability experiences of the product subjectively [44].

First-person surveys in User Research | by Nikki Anderson | UX Collective. [cited 27 Oct 2022]. Available: https://uxdesign.cc/first-person-surveys-in-user-research-8732c5d9ab96

10. According to what?

We thank the reviewer's comment. We have clarified this information by adding a new sentence according to the comment. Please see page 14, line 272, and below

Once the questionnaire was finished, ten users (see demographic characteristics in table 5) read and answered the questionnaire to know if all the requirements from the Delphi study were met.

11. It was a criterium for inclusion... Not here...

Following the reviewer's advice, we have removed this part from the text.

12. Initially the authors raised my expectation that they would develop a simple questionnaire to be used by people with neurological diseases and that there were no such resources adapted to you. How does this questionnaire differ from others?

We agree with the reviewer's comment. This questionnaire has been created from the responses of both health professionals specialized in neurology and patients with neurological disorders, so it has been designed based on a user-centered design, unlike the questionnaires that currently exist for the same purpose. In addition, the existing questionnaires, as I have already mentioned in the article, are either very long or include questions/items that are not relevant for all countries, as is the case with QUEST, which includes a part of after-sales service and technical issues that many people with diseases of neurological origin would not know how to respond. In addition, our questionnaire has included the relevant items for these people and why they decide to use or not use the AT product.

13.How does it differ from the health conditions that led to the other assistive technology tools?

We appreciate the reviewer's comment. In the sentence, we would like to state that in the study, we analyze different tools which have been analyzed by different people with different neurological conditions. Moreover, for these reasons, the sample has good heterogeneity.

REVIEWER 2

1.Can the authors elaborate a bit more the relationship between AT selection and outcome assessment?

Following your advice, we have added some sentences addressing your question. Please see page 4, line 81, and below:

For example, professionals prescribe assistive products without considering the user's needs. However, this clinical outcome assessment is important in clinical practice and research

because it improves evidence and provides considerable feedback to healthcare professionals and patients, enhancing their empowerment with their opinions and needs and improving their quality of life[16].

2. Do you mean "usability of AT?". I would suggest you to add the word "usability" in this sentence.

When I read any paper on usability, I always would like to see a definition of usability. if you have space, please add a definition of usability so the readers would know "what usability" are you focusing on since you mentioned the QUEST's technical items are difficult so your questionnaire does not focus on technical part.

We appreciate the reviewer's comment. We have added the Usability term and its definition. Please see page 3, line 68 and below:

According to the ISO 9241-11 [9], usability is the extent to which specified users can use a product to achieve specified goals with effectiveness, efficiency, and satisfaction in a specified context of use. Understanding this, the design of a product…

3. I got a feeling some words are missing here. Please rephrase the sentence

We appreciate the reviewer's comment. We have rewritten the sentence. Please see page 4, line 91, and below:

There are other questionnaires developed exclusively to assess wheelchairs[22], and only a few are explicitly developed for assistive technologies. For example, the Quebec User Evaluation of Satisfaction with Assistive Technology (QUEST 2.0) [23] contains technical items such as weight, product dimensions, service delivery, repairs, and device services…

4. Can the authors clarify what you meant by limitations in the accessibility?

Following the reviewer's advice, we have modified the sentence. Please see page 5, lines 95 and below:

People with neurological diseases have limitations in the accessibility of the existing usability questionnaires, for example, in the comprehension of the questions or the answer form.

5. The recruitment of end users is an important part in this study. Now it is missing in the method. Please add in the recruitment process of end users.

We appreciate the reviewer's comment and have added a new sentence with this information. Please see page 5, line 114, and below:

Also, all the patients from the Institut Guttmann who voluntarily agreed to participate were interviewed to answer the questionnaire.

6. CAn you the authors elaborate what these values are or what they represent.

Following the reviewer's suggestion, we have added a new sentence. Please see page 7, line 141, and below:

Subsequently, the users (people with neurological diseases) read the questionnaire to assess their understanding of the questions and if their answers could be represented with the proposed response values (numbers, traffic light colors, and faces).

7. I do not understand this sentence and how this sentence is related to the sample. Do the participants have to pass the SPMSQ test in order to be eligible to fill in the questionnaire? Please rephrase the sentence.

Following the reviewer's suggestion, we have added a new sentence. Please see page 7, line 148, and below:

Exclusion criteria moderate to severe cognitive impairment based on the Pfeiffer SPMSQ test [32] translated into Spanish[33].

8. since you only use ref 33, it is more appropriate to mention the authors' last names and not just several authors.

We thank the reviewer's observation; we have corrected it and added the missing reference. Please see page 8, line 165, and below

According to different authors,[35,36] an interval between two days and two weeks between the test-retest interviews is recommended.

9. Please add which ICC model you have used, such as ICC 2.1 , ICC 3.1 etc. Please add it here and also in the results

We thank the reviewer's remark. According to this remark, we have added a new sentence. Please see page 8, line 172, and below.

Intraclass correlation coefficient (ICC) model 1.1 with a 95% of confidence interval (CI) was measured.

10. Please elaborate to your readers what do you mean by behaviour of items in the context of validity.

Following the reviewer's suggestion, we have added a new sentence. Please see page 9, line 190, and below:

In order to further analyze the construct validity of the different items, the Rasch model [41] was carried out.

11. this part of the analysis is an analysis of concurrent validity. Construct validity is an umbrella term for different validities.

My suggestion is to change this term to "concurrent validity".

Thank you for your valuable observation. Following the reviewer's suggestion, we have modified the term. Please see page 8, line 181, and below:

Concurrent validity. To know the relationship between the new questionnaire and another related questionnaire, QUEST 2.0[23] was chosen

12 . which model are you using? rating scale model?

We thank the reviewer's observation. We are using the Rasch model because it is commonly used in the analysis of the construct validity of an instrument. Please see the reference:

Boone WJ. Rasch Analysis for Instrument Development: Why, When, and How? CBE Life Sci Educ. 2016;15. doi:10.1187/CBE.16-04-0148

13. Move this sentence to the previous parapraph

Following the reviewer's suggestion, we have moved the sentence to the previous paragraph. Please see page 9, line 196 and below:

The study was developed following the COSMIN guidelines[42].

Ethical Approval

14. even though it is only 2 persons, it is not valid to ask end users that do not have any AT to answer the questions in the Delphi study, such as "would you change anything about our existing products?".

We thank the reviewer's observation. These patients didn't use the AT at the moment of the interview; however, they have used it in the past (last weeks, months)
We will add a sentence to clarify it. Please see table 1 on page 11 and below:

[c]Nothing means that he/she did not use assistive technologies at the moment of the interview; however, they used it in the past

15.Did these experts involve in the rounds? Please clarify.

Following the reviewer's advice, we have added a new sentence. Please see page 12, line 232, and below

Thirty-four experts from the Delphi study from the private neurorehabilitation hospital of acute neurological patients and the public hospital of acute stroke patients were selected to evaluate the items obtained from round two.

16. Ergonomics is related to body posture and positions, often in workplace. My daily live needs is much bigger than body posture. Please explain why this item is categorized as ergonomics?

We thank the reviewer for this suggestion. According to the ISO 9241-11:2018(en), Ergonomics of human-system interaction — Part 11: Usability: Definitions and concepts, ergonomics/human factor is a scientific discipline concerned with the understanding of interactions among humans

and other elements of a system, and the profession that applies theory, principles, data, and methods to design in order to optimize human well-being and overall system performance,

Another definition: Ergonomics is the design and engineering of human-machine systems to enhance human performance.

Dempsey, Patrick G., Wogalter, Michael S., & Hancock, Peter A. (2000). What is in a name? Using terms from definitions to examine the fundamental foundation of human factors and ergonomics science. Theoretical Issues in Ergonomics Science, 1(1), 3-10.

Also, see the text below from Ahasan, R., Campbell, D., Salmoni, A., & Lewko, J. (2001). HFs/ergonomics of assistive technology. Journal of physiological anthropology and applied human science, 20(3), 187–197. https://doi.org/10.2114/jpa.20.187.

How ergonomic designs could be helpful for special needs, Coleman and Pullinger (1993) noted some aspects of improvement features of disability and the aging process. Ergonomics application is important in the design and development of AT towards meeting the needs of disabled people. Hence by addressing the actual needs of these people, ergonomics principles are to be followed to enhance the mobility and communication of disabled people. Ergonomically designed AT usually enhance functional requirements that correctly fit the future application of the users meeting the needs of disabled people.

For these reasons, we thought that ergonomics fit in this category.

17. Please provide information about these ten users? were they in the Delphi rounds? do you do AT? what are their diagnoses?

We appreciate the reviewer's comment. We have added a new sentence in order to clarify it. Please see page 14, line 271.

Once the questionnaire was finished, ten users (see demographic characteristics in table 5)

Also, we have added a new table with the demographic data of the ten users. Please see page 15, line 274, and below:

Table 5. Demographic characteristics users from the questionnaire design.

Questionnaire analysis	n= 10	
**Demographic Characteristics**	**Number**	**Percentage**
**Age**		
Between 18 and 25 years	n=1	10%
Between 26 and 35 years	n=2	20%
Between 36 and 45 years	n=2	20%
Between 46 and 65 years	n= 3	30%
More than 65 years	n= 2	20%
**Sex**		
Female	n=6	60%
Male	n=4	40%
**Pathology**		
Spinal Cord Injury	n=4	40%
Traumatic Brain Injury	n= 2	20%
Stroke	n= 3	30%
Other	n= 1	10%
**Assistive Technologies**		
Wheelchair	n= 8	80%
Canes	n= 1	10%
Orthesis	n=4	40%
Other	n=1	10%
Nothing[a]	n=1	10%

[a]Nothing means that he/she did not use assistive technologies at the moment of the interview; however, they used it in the past

18. What is a usability test? please clarify

We thank the reviewer's observation, and we have added a new sentence in the introduction to clarify it. Please see page 4, line 73 and below:

For that reason, it is essential to perform a usability test during product development to know if it fits the user's needs. Usability testing refers to evaluating a product or service by testing it with representative users [11].

19. did these 51 patients not the end users in the Delphi study? if not, please replace the word "round" with " adminstration" or " occasion".

We agree with the reviewer's comment. We have changed the word "round" to "administration" to distinguish. Also, we expressed that the patients are different from the Delphi study. Please see page 16, line 285, and below:

A total of 51 people with neurological diseases consecutive recruited from the Institut Guttmann Hospital voluntarily agreed to participate in the study. These people were different from the Delphi study, and their demographic characteristics are summarized in Table 6.

20. Brain injury is often acquired or traumatic. What are the brain injuries in these 4 patients?

We thank the reviewer's observation, and we have corrected it. Please see table 6 on page 16 and below:

Table 6. Demographic characteristics of the 51 participants in the reliability and validity of NATU Quest.

**Demographic Characteristics**	**Mean /Number**	**Percentage**
Age, range 16-72 years	Mean = 48	
**Sex**		
Female	n=20	39%
Male	n= 31	61%
**Pathology**		
Spinal cord Injury	n=22	43%
Traumatic Brain Injury	n=3	6%
Stroke	n=16	31%
Acquired Brain injury	n=4	8%
Other neurological diseases	n=6	12%
**Education**		
Secondary	n=13	25%
High School Diploma	n=1	2%
Certificate of Professional Standards	n=15	29%
Higher Level Education Cycle	n=7	14%

University Degree	n=15	30%
**Questionnaire form**		
Self-administration	n=24	53%
Interview	n=27	47%
**Assistive Technologies**		
Wheelchair	n=29	
Walking stick	n=8	
Walker	n=3	
Splint	n=7	
Treadmill	n=5	

21.Cognitive impairments are common in persons with neurological diseases. Do you have a criterion that the end users do not have cognitive impairments?

And Pfeiffer test was not mentioned at all in the method. Please add this test in the method section.

We appreciate the reviewer's comment. Pfeiffer test was mentioned in the inclusion/exclusion criteria. Please see page 7, line 148, and below:

Exclusion criteria moderate to severe cognitive impairment based on the Pfeiffer SPMSQ test [32] translated into Spanish[33].

22.a question about the expertise of doctors and PTs in assistive technology. Do doctors work with AT ? it is often occupational therapists and physiotherapists who have knowledge. Please add more information about doctors' expertise in AT in your country.

We thank the reviewer's comment. We have exchanged medical doctors with occupational therapists to make it more understandable. However, in the center where we performed the study, there is a transdisciplinary team, and the prescription of the orthoses is agreed upon by medical doctors, PT, occupational therapists, and orthopaedists. Please see page 18, line 325 and below:

The items included in the questionnaire, the format of the questionnaire, and the answer form were derived through two rounds of a Delphi study[27] based on the opinion of 69 experts (neurorehabilitation professionals, such as occupational therapists and physiotherapists) and 63 users (people with neurological diseases).

23. do you know if the other regions in Spain have similar experiences?

Following the reviewer's suggestion, we have added a new sentence. Please see page 19, line 343, and below:

(1) Selection bias of the participants since most of them were from the same province. However, other regions have the same experiences. [45]

24. no information on Pfeiffer test in the study.

We appreciate the reviewer's comment. Pfeiffer test was mentioned in the inclusion/exclusion criteria. Please see page 7, line 148, and below:

Exclusion criteria moderate to severe cognitive impairment based on the Pfeiffer SPMSQ test [32] translated into Spanish[33].

25. the ATs in your study are mobility AT. Do you want to measure only mobility AT or other AT that support cognition? if yes, please add more information.

We thank the reviewer's observation. We only wanted to measure mobility AT. Others, such as cognition or audition, have other parameters and items.

26. I don't see if your test-retest reliability has compared with Quest 2. Please clarify.

We thank the reviewer's observation. Please see the results from pages 15 to 17 and in table 7 and below:

Reliability and Validity

Sample description

A total of 51 people with neurological diseases consecutive recruited from the Institut Guttmann Hospital voluntarily agreed to participate in the study. These people were different from the Delphi study, and their demographic characteristics are summarized in Table 6. Fifty-three percent of the participants answered the questionnaire through an interview due to physical

limitations, and the rest (47%) answered it by themselves. First, the participants answered the new questionnaire and the QUEST 2.0. On average, the participants completed the new questionnaire within 102.40 seconds in the first administration and within 82.08 seconds in the second administration. The QUEST 2.0 was administered once before the NATU Quest, and the participants needed an average of 74 seconds to complete it. Participants scored an assistive product they had used in the last three months. All the patients answered all the items.

Table 6. Demographic characteristics of the 51 participants in the reliability and validity of NATU Quest.

**Demographic Characteristics**	**Mean /Number**	**Percentage**
Age, range 16-72 years	Mean = 48	
**Sex**		
Female	n=20	39%
Male	n= 31	61%
**Pathology**		
Spinal cord Injury	n=22	43%
Traumatic Brain Injury	n=3	6%
Stroke	n=16	31%
Acquired Brain injury	n=4	8%
Other neurological diseases	n=6	12%
**Education**		
Secondary	n=13	25%
High School Diploma	n=1	2%
Certificate of Professional Standards	n=15	29%
Higher Level Education Cycle	n=7	14%
University Degree	n=15	30%
**Questionnaire form**		
Self-administration	n=24	53%
Interview	n=27	47%
**Assistive Technologies**		
Wheelchair	n=29	
Walking stick	n=8	
Walker	n=3	
Splint	n=7	

Treadmill	n=5	

Reliability Results

The internal consistency reliability of the NATU Quest was analyzed using the Cronbach's Alpha[34] (α=0.895). This result can be interpreted as good reliability.

Reliability through test-retest. A retest was performed 15 days after answering the two questionnaires for the first time to assess the reliability of the NATU Quest. Table 7 shows the weighted quadratic Kappa coefficient and Spearman's coefficient results of the NATU Quest. The results showed a moderate to considerable concordance between NATU Quest items test-retest in the Kappa coefficient because all the results were above 0,50. The results also showed a strong association between test-retest in the Spearman's coefficient (ρ = 0.818), significant with p-value < 0.0001. The results of the ICC showed good reliability (ICC = 0.869; CI 95% 0.781 to 0.923).

Table 7. NATU Quest reliability of the test-retest.

**NATU Quest**				
**Items**		**Weighted Kappa**	**95% Confidence Interval**	
			**Lower Limit**	**Upper Limit**
1	Effectiveness	0.727	0.535	0.918
2	Comfortable	0.733	0.645	0.821
3	Adaptability	0.585	0.434	0.735
4	Easy to put on/off	0.631	0.399	0.863
5	Safe	0.551	0.206	0.895
6	Functionality	0.685	0.541	0.828
7	Ergonomics	0.673	0.507	0.840
8	Easy to use	0.544	0.332	0.755
9	Easy to remember how to use it	0.624	0.298	0.950
10	Satisfaction	0.714	0.557	0.871
**Spearman**		0.818[a]		

[a]The correlation is significant p< 0.0001 (bilateral).

Concurrent validity

The correlation of the total scores between NATU Quest and QUEST 2.0 analyzed with the Spearman's coefficient was strong with $\rho = 0.756$ significant, with p-value < 0.0001

In the name of the authors, I hope that this revised manuscript answers all the concerns contained in the reviews, and we are grateful for the thought and effort the reviewers have put into these reviews.

Yours sincerely.

Francesc Rubí-Carnacea
