# Supplementary material for: Developing an assistive technology usability questionnaire for people with neurological diseases
Source: PLoS One. 2023 Jan 31;18(1):e0281197. doi: 10.1371/journal.pone.0281197 (PMC9888697; doi:10.1371/journal.pone.0281197)
Supplement: Attachment — Submitted filename: Response to Reviewers4.docx [file pone.0281197.s007.docx]

**Dear Editor of PLOS ONE,**

I am pleased to resubmit the revised version of PONE-D-22-13676 " Developing An Assistive Technology Usability Questionnaire for People with Neurological Diseases" for your consideration. We are very grateful for the excellent suggestions and comments from the reviewers. As you suggested, we have carefully considered all the suggestions and comments and made revisions accordingly. Thanks again for those valuable suggestions, which have helped us further improve the quality and clarity of this manuscript. We have addressed each reviewer's requirements as outlined below, which are indicated in the manuscript in yellow.

**REVIEWER 1**

In reference to the comments to reviewer 1, we apologize if he/she thinks that we did not answer the questions. Please see the answers below:

**1. Assistive Technologies, instead of Nervous System Diseases????**

We thank the reviewer's observation. We have added “Assistive technologies” instead of “Nervous System Diseases”. Please see page 2, line 47, and below:

“Keywords: Assistive technologies, Neurological Rehabilitation, Questionnaire Design, Self-Help Devices, User-centered Design”

**2. functioning, instead**

Thank you for the comment. According to the comment, we have changed the word functionality to functioning. Please see page 2, line 59 and below:

“improve their functioning and autonomy in their daily life.”

Also, see page 10 line 211 and below:

“Round two involved 59 people (27 experts and 32 users) (Table 1). This round obtained 15 items and data about the scale. The items were: "effectiveness", "comfort", "adaptability", "easy to put on/off", "safe", "lightweight", "functioning", "ergonomic", "economical", "affordable", "easy to use", "feedback", "stimulating," "monitored" and "movement facilitator.”

Also see page 18 line 331 and below:

“The item "functioning" appear in the USAT-WM.[22]”

**3. This definition is very questionable! The authors have taken and written ipsis verbis from [10] who, in turn, took it from Fed Regist. 1991;56(60):4121... 1991!!!!!!!!!!!!!!!!! It will be useful to reformulate this text and, consequently, the conceptual framework of this definition.**

Thank you very much for your comment. The definition was too old, so we have added a new definition. Please see page 4, line 76 and below.

“According to the WHO, assistive technology is a general term covering the systems and services related to delivering assistive products and services.[12] Assistive products aim to maintain or improve an individual's functioning and independence, promoting their well-being.[12]”

**4. To use the term "person with neurological diseases" is not entirely correct. I think the authors want to develop a tool to assess the usability of assistive technology products and user satisfaction and make it accessible to everyone, including those with communication limitations/restritions, cognitive/mental deficits/etc. It would not be appropriate to talk about "neurological disorders" in general, as there are some persons with neurological diseases, but who do not present any difficulty in understanding and answering the existing questionnaires.**

We appreciate the reviewer's comment; however, this questionnaire has been developed for people with neurological diseases. The authors do not aim to develop a tool to assess the usability of assistive technology for other patients´ typologies.

**5. Why did you use the "Short Portable Mental Status Questionnaire for the Assessment of Organic Brain Deficit in Elderly Patients", since it is an instrument for the elderly population and the sample used ranged from 16 to 72 years (mean age 48 years)?**

Thank you for your valuable observation. We agree with the reviewer's comment. We chose the Pfeiffer test instead of MMSE because it is shorter and more manageable for these patients. We included this point in the limitations section. Please see page 19, line 344, and below:

“2) Although all the users who participated in the validation had a neurological disease, the authors chose the Pfeiffer SPMSQ to assess the cognitive problems because it is short and quick to answer. However, Pfeiffer SPMSQ does not accurately assess all possible cognitive deficits, and it is not sensitive enough to detect low or mild cognitive deficits.”

**6.Don't you think it is too short??!! Aren't the users still learning how to dela with the assistive device?**

We appreciate the comment. We chose at least one month for practical reasons because we performed the study in a hospital where most patients are acute and subacute. In addition, other usability questionnaires, such as PIADS and QUEST, do not express this point.

Moreover, we have added this to the limitations section. Please see page 19, line 351, and below:

“(4) For practical reasons we chose that the users have used the product at least for one month, however, probably is not enough time to test a product.”

**7. Explanation?**

We appreciate the reviewer's comment. These patients did not use the AT at the moment of the interview; however, they used it in the past (last weeks, months)

Following the reviewer's comment, we have added a sentence to clarify it. Please see table 1, pages 10 and 11, and table 5, page 15 and below:

“Nothing means that he/she did not use assistive technologies at the moment of the interview; however, she/he used them in the past.”

**8. Why from the acute?**

We appreciate the reviewer's comment. In the study, we included acute, subacute and chronic patients, however, in order to avoid misunderstandings we have deleted hospitals’ characteristics.

Please see page 5, line 112 and below:

“A google form questionnaire was sent via email to the neurological healthcare professionals from the Institut Guttmann, Spain and Hospital de l’Esperança, Spain.

Please see page 11, line 230 and below:

“Thirty-four experts from the Delphi study from the Institut Guttmann and the Hospital de l'Esperança were selected to evaluate the items obtained from round two..”

**9. Read the questionnaire to assess their understanding of the questions and if their answers could be represented with the proposed response.**

Thank you for your appreciation. We have added a new sentence in order to clarify the sentence. Please see page 6, line 137 and below:

“Subsequently, ten users (people with neurological diseases) read the questionnaire to assess their understanding of the questions and if their answers from the Delphi study could be represented with the proposed response values (numbers, traffic light colors, and faces).”

**10. Do you search for references/support? Please use a Reference.**

Thank you for your observation. Following your advice, we have added a new reference. Please see page 14, line 268, and below:

“The statements of the questions are in first person to facilitate users' answers and usability experiences of the product subjectively [44].”

First-person surveys in User Research | by Nikki Anderson | UX Collective. [cited 27 Oct 2022]. Available: https://uxdesign.cc/first-person-surveys-in-user-research-8732c5d9ab96

**11. According to what?**

We thank the reviewer's comment. We have clarified this information by adding a new sentence according to the comment. Please see page 14, line 268, and below:

“Once the questionnaire was finished, ten users (see demographic characteristics in table 5) read and answered the questionnaire to know if all the requirements from the Delphi study were met.”

**12. It was a criterium for inclusion... Not here...**

Thank you for your comment. Following the reviewer's advice, we have removed this part from the text.

**13. Initially the authors raised my expectation that they would develop a simple questionnaire to be used by people with neurological diseases and that there were no such resources adapted to you. How does this questionnaire differ from others?**

Thank you for your comment. As we have already mentioned in the article, the existing questionnaires are either very long or include questions/items that are not relevant for all countries, as is the case with QUEST, which includes a part of after-sales service and technical issues that many people with diseases of neurological origin would not know how to respond. In addition, our questionnaire has included the relevant items for these people and why they decide to use or not use the AT product.

**14.How does it differ from the health conditions that led to the other assistive technology tools?**

We appreciate the reviewer's comment. The questionnaire was designed by people with neurological diseases following their particular needs. As we mentioned in future work, validating this questionnaire with a different population would be interesting.

**REVIEWER 2**

**1.I agree with your two references and my suggestion is to change item 7: ________adapts to my special needs. My daily live needs is broader than my special needs.**

**As your reference said "....for special needs" and this instrument is about AT for persons with neurological illnesses,**

We agree with the reviewer's comment. Following the reviewer's suggestion, we have changed item 7. Please see pages 13 and 14, table 4, and below:

**Table 4. Questions and the items that are involved in each question.**

| **Questions** | | **Items** |  |
| --- | --- | --- | --- |
| 1 | I believe that ­_____ can help me improve my functional independence | Effectiveness | |
| 2 | I feel comfortable wearing/using ____ | Comfortable | |
| 3 | _____ adapts to my characteristics and needs | Adaptability | |
| 4 | Donning/Doffing …………….. is quick and easy for me. | Easy to put on / off | |
| 5 | I feel safe using/wearing _____ / _____ is safe in its use | Safe | |
| 6 | _____ allows me to achieve my goal/ allows me to perform a movement/action that I could not do before | Functionality/ movement facilitator | |
| 7 | _____ adapts to my special needs. | Ergonomics | |
| 8 | In general, _____ is easy to use | Easy to use | |
| 9 | Information and instructions of use______ are easy to understand and easy to remember | Easy to remember how to use it | |
| 10 | Overall, I am satisfied with______ | Satisfaction | |

**2. what does SPMSQ stand for?**

We have clarified this information by adding a new sentence. Please see page 7, line 146, and below:

“Exclusion criteria moderate to severe cognitive impairment based on the Pfeiffer Short Portable Mental State Questionnaire (Pfeiffer SPMSQ).”

**3.since it is only 1 person, do not use the word "they" but he/she**

We appreciate the reviewer's observation, and we have corrected it. Please see table 1, page 11 and line 224; and table 5, page 15, line 273, and below:

“Nothing means that he/she did not use assistive technologies at the moment of the interview; however, she/he used it in the past.”

-----

“In the second round, 36% did not answer the questionnaire. Others are, for example, orthopedic professionals and engineers. Nothing means that he/she did not use assistive technologies at the moment of the interview; however, she/he used them in the past. Users are different from round 1.”

In the name of the authors, I hope that this revised manuscript answers all the concerns contained in the reviews, and we are grateful for the thought and effort the reviewers have put into these reviews.

Yours sincerely.

Francesc Rubí-Carnacea
