# Supplementary material for: Correction: Developing an assistive technology usability questionnaire for people with neurological diseases
Source: PLoS One. 2024 Aug 2;19(8):e0308511. doi: 10.1371/journal.pone.0308511 (PMC11296618; doi:10.1371/journal.pone.0308511)
Supplement: S2 File — (PDF) [file pone.0308511.s001.pdf]

S2 File. NATU Questionnaire, scoring and interpretation.

NATU Quest - Spanish version

Producto que evaluamos:
……………………………………… (Por favor añade el nombre del producto)

Responde las preguntas utilizando la escala del 0 al 5:

- 0 Totalmente en desacuerdo
- 5 Totalmente de acuerdo

1. Creo que ………… puede ayudarme a mejorar mi independencia funcional.

0	1	2	3	4	5

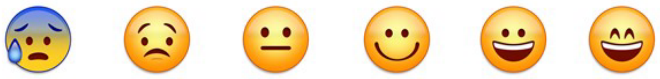

Comments:

2. Me siento cómodo usando/llevando……………………….

0	1	2	3	4	5

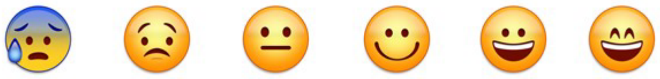

Comments:

3. …………………….se adapta a mis características y necesidades.

0	1	2	3	4	5

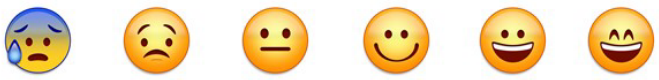

Comments:

4. Es rápido y fácil poner/quitar…………..

0	1	2	3	4	5

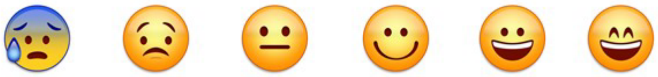

Comments:

5. Me siento Seguro usando/llevando…………// ………………… es seguro en su utilización.

0	1	2	3	4	5

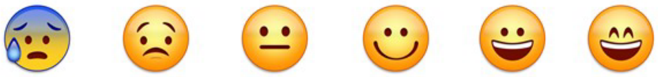

Comments:

6. ……………………….. me permite conseguir mi objetivo // me permite realizar un movimiento/acción que antes no podía hacer.

0	1	2	3	4	5

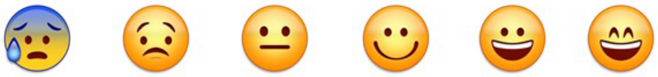

Comments:

7. …………………………….. se adapta a mí en mis necesidades de la vida diaria.

0	1	2	3	4	5

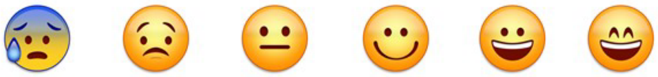

Comments:

8. En general, ………………………………. es fácil de usar.

0 1 2 3 4 5

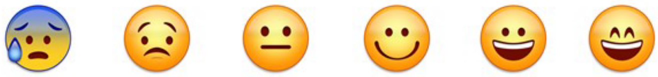

Comments:

9. La información e instrucciones dadas para usar …………………………. son fáciles de entender y recordar.

0 1 2 3 4 5

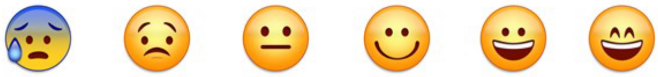

Comments:

10. En general, estoy satisfecho/a con …………………

0 1 2 3 4 5

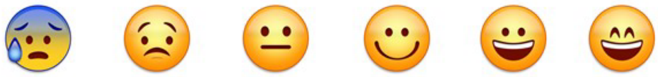

Comments:

Resultados

TOTAL

Usable

Ligeramente Usable

No Usable

Eficacia

Comodidad

Adaptabilidad

Fácil de poner y quitar

Seguro

Funcionalidad

Ergonomía

Fácil de usar

Fácil de recordar como se usa

Satisfacción

Puntuación e interpretación del cuestionario

La puntuación total del cuestionario se obtuvo sumando las puntuaciones de las respuestas válidas del 1 al 10 y dividiendo la suma por el número de ítems válidos. Este valor varía de 0 a 5.

Interpretación de resultados: Dado que el cuestionario es una escala tipo Likert de 6 los valores de 0 a 2 son resultados negativos y de 3 a 5 son resultados positivos, siguiendo estas premisas:

- *Usable* Si la puntuación total fue de 4 a 5, indica que el usuario está satisfecho con el producto y es probable que lo utilice. Además, si el producto era un prototipo o en desarrollo, el resultado indica que el dispositivo cumple con los requisitos mínimos para ser utilizable y no necesita revisión o necesita muy poca revisión de algunos ítems.
- *Ligeramente usable*. Si el resultado fue ≥ 3, significa que el usuario no está completamente satisfecho y puede dejar de usarlo. Además, si el producto estaba en desarrollo, significa que el dispositivo cumple con los requisitos mínimos para ser utilizable, pero requiere revisión de algunos ítems.
- *No usable.* Si el resultado fue < 3, significa que el usuario no está completamente satisfecho e inevitablemente dejará de usarlo. Además, si el producto está en desarrollo, indica que el dispositivo no cumple con los requisitos mínimos para ser utilizable y requiere revisión de varios ítems o necesita ser reconsiderado.

Es posible obtener una puntuación para cada ítem con el fin de proporcionar una indicación más precisa de qué elementos necesitan una mejora en caso de que se esté desarrollando una nueva tecnología de asistencia. Para calcularlo se suman todas las puntuaciones válidas de cada ítem de todos los cuestionarios contestados y se divide la suma por el número de respuestas válidas de todos los cuestionarios, de ahí se obtiene una puntuación media para cada ítem.

- Si la puntuación obtenida fue 5, indica que el ítem del producto abordado no requiere revisión alguna.
- Si la puntuación obtenida fue < 5 y ≥ 3, indica que este ítem del producto requiere revisión.
- Si la puntuación fue < 3, este ítem del producto requiere una revisión exhaustiva.
